# The MedSafer Study—Electronic Decision Support for Deprescribing in Hospitalized Older Adults: A Cluster Randomized Clinical Trial

**DOI:** 10.1001/jamainternmed.2021.7429

**Published:** 2022-01-18

**Authors:** Emily G. McDonald, Peter E. Wu, Babak Rashidi, Marnie Goodwin Wilson, Émilie Bortolussi-Courval, Anika Atique, Kiran Battu, Andre Bonnici, Sarah Elsayed, Allison Goodwin Wilson, Louise Papillon-Ferland, Louise Pilote, Sandra Porter, Johanna Murphy, Sydney B. Ross, Jennifer Shiu, Robyn Tamblyn, Rachel Whitty, Jieqing Xu, Gabriel Fabreau, Taleen Haddad, Anita Palepu, Nadia Khan, Finlay A. McAlister, James Downar, Allen R. Huang, Thomas E. MacMillan, Rodrigo B. Cavalcanti, Todd C. Lee

**Affiliations:** 1 Division of General Internal Medicine, Department of Medicine, McGill University Health Centre, Montreal, Quebec, Canada; 2 Clinical Practice Assessment Unit, Department of Medicine, McGill University Health Centre, Montreal, Quebec, Canada; 3 Division of Experimental Medicine, Department of Medicine, McGill University, Montreal, Quebec, Canada; 4 Division of Clinical Pharmacology & Toxicology, Department of Medicine, University of Toronto; Division of General Internal Medicine and Geriatrics, University Health Network, Toronto, Ontario, Canada; 5 Division of General Internal Medicine, Department of Medicine, University of Ottawa, Ottawa, Canada; 6 Division of General Internal Medicine, Department of Medicine, University of British Columbia, Vancouver, British Columbia, Canada; 7 Faculty of Medicine and Health Sciences, McGill University, Montreal, Quebec, Canada; 8 Department of Pharmacy, University Health Network, Toronto, Ontario, Canada; 9 Department of Pharmacy, McGill University Health Centre, Montreal, Quebec, Canada; 10 Faculty of Pharmaceutical Sciences, University of British Columbia, Vancouver, British Columbia, Canada; 11 Department of Pharmacy, Institut Universitaire de Geriatrie de Montreal, University of Montreal, Montreal, Quebec, Canada; 12 Division of Epidemiology, Department of Medicine, McGill University Health Center, Montreal, Quebec, Canada; 13 Division of General Internal Medicine, Department of Medicine, Queen’s University, Kingston, Ontario, Canada; 14 Alberta Health Services, Edmonton, Alberta, Canada; 15 Leslie Dan Faculty of Pharmacy, University of Toronto, Toronto, Ontario, Canada; 16 Department of Medicine, The University of Ottawa, Ottawa, Ontario, Canada; 17 Division of General Internal Medicine, University of Calgary, Calgary, Alberta, Canada; 18 Division of Geriatric Medicine, Queens University, Kingston, Ontario, Canada; 19 Division of General Internal Medicine, University of Alberta Hospital, Edmonton, Alberta, Canada; 20 Division of Palliative Care, University of Ottawa, Ottawa, Ontario, Canada; 21 Division of Geriatric Medicine, Department of Medicine, The Ottawa Hospital, Ottawa, Ontario, Canada; 22 Division of General Internal Medicine, Department of Medicine, University of Toronto, Toronto, Ontario, Canada; 23 HoPingKong Centre for Excellence in Education and Practice, University Health Network, Toronto, Ontario, Canada; 24 Division of infectious Diseases, Department of Medicine, McGill University Health Centre, Montreal, Quebec, Canada

## Abstract

**Importance:**

Scalable deprescribing interventions may reduce polypharmacy and the use of potentially inappropriate medications (PIMs); however, few studies have been large enough to evaluate the impact that deprescribing may have on adverse drug events (ADEs).

**Objective:**

To evaluate the effect of an electronic deprescribing decision support tool on ADEs after hospital discharge among older adults with polypharmacy.

**Design, Setting, and Participants:**

This was a cluster randomized clinical trial of older (≥65 years) hospitalized patients with an expected survival of more than 3 months who were admitted to 1 of 11 acute care hospitals in Canada from August 22, 2017, to January 13, 2020. At admission, participants were taking 5 or more medications per day. Data analyses were performed from January 3, 2021, to September 23, 2021.

**Interventions:**

Personalized reports of deprescribing opportunities generated by MedSafer software to address usual home medications and measures of prognosis and frailty. Deprescribing reports provided to the treating team were compared with usual care (medication reconciliation).

**Main Outcomes and Measures:**

The primary outcome was a reduction of ADEs within the first 30 days postdischarge (including adverse drug withdrawal events) captured through structured telephone surveys and adjudicated blinded to intervention status. Secondary outcomes were the proportion of patients with 1 or more PIMs deprescribed at discharge and the proportion of patients with an adverse drug withdrawal event (ADWE).

**Results:**

A total of 5698 participants (median [range] age, 78 [72-85] years; 2858 [50.2%] women; race and ethnicity data were not collected) were enrolled in 3 clusters and were adjudicated for the primary outcome (control, 3204; intervention, 2494). Despite cluster randomization, there were group imbalances, eg, the participants in the intervention arm were older and had more PIMS prescribed at baseline. After hospital discharge, 4989 (87.6%) participants completed an ADE interview. There was no significant difference in ADEs within 30 days of discharge (138 [5.0%] of 2742 control vs 111 [4.9%] of 2247 intervention participants; adjusted risk difference [aRD] −0.8%; 95% CI, −2.9% to 1.3%). Deprescribing increased from 795 (29.8%) of 2667 control to 1249 (55.4%) of 2256 intervention participants [aRD, 22.2%; 95% CI, 16.9% to 27.4%]. There was no difference in ADWEs between groups. Several post hoc sensitivity analyses, including the use of a nonparametric test to address the low cluster number, group imbalances, and potential biases, did not alter study conclusions.

**Conclusions and Relevance:**

This cluster randomized clinical trial showed that providing deprescribing clinical decision support during acute hospitalization had no demonstrable impact on ADEs, although the intervention was safe and led to improvements in deprescribing.

**Trial Registration:**

ClinicalTrials.gov Identifier: NCT03272607

## Key Points

**Question:**

Does providing clinical decision support during an acute care hospitalization improve deprescribing of potentially inappropriate medications and 30-day postdischarge adverse drug events (ADEs) in older adults?

**Findings:**

This cluster randomized multicenter trial of 5698 hospitalized participants found that providing electronically generated deprescribing reports did not have a significant impact on ADEs within 30 days despite increased deprescribing at discharge.

**Meaning:**

The findings of this randomized clinical trial indicate that clinical decision support during hospitalization improves deprescribing but has little impact on medication harms in the short term.

## Teaser

This randomized clinical trial examines the impact of electronic decision support on postdischarge adverse drug events and deprescribing among hospitalized patients 65 years or older at 11 hospitals in Canada.

## Introduction

Deprescribing is the medically supervised tapering or cessation of medications that are no longer needed or beneficial, including medications that are potentially inappropriate (PIMs), time delimited, ineffective, or that do not align with goals of care.^1^ After an acute care hospitalization, adverse drug events (ADEs) are common and up to 60% are considered preventable.^2^ Deprescribing PIMs at hospital discharge to prevent ADEs could be advantageous; however, several barriers exist.^3,4^ First, hospital practitioners focused on acute care requirements may be reluctant to alter long-term outpatient therapies managed by other prescribers, particularly if they do not follow patients postdischarge.^5^ Second, not all centers include routine pharmacist or geriatrician subspecialties to support interprofessional care. Third, some prescribers fear that stopping or reducing a drug can lead to adverse effects.^6^ Fourth, despite deprescribing opportunities, therapeutic inertia is common^7,8^; true even for low-risk drugs with no withdrawal concerns^9^ and more so for drugs with higher risks of harm.^10^ Even when adverse events occur, opportunities to deprescribe are not always recognized or acted on.^11,12^ To our knowledge, prior studies of interventions to reduce PIMs while in hospital^13,14,15^ have tended to be underpowered and nonrandomized or did not capture postdischarge ADEs.

We previously developed and tested an electronic decision support tool,^16^ MedSafer, in a pilot controlled before and after study of deprescribing in the acute care setting. MedSafer integrates data available in the electronic health record (EHR)^17^ to generate reports with evidence-based deprescribing opportunities, stratified according to prespecified expert consensus of perceived drug risks. In our pilot study, providing MedSafer reports to practitioners increased the absolute rate of PIM deprescribing by 8.3%.^16^ The primary objective of the present study was to perform a large stepped-wedge cluster randomized trial to evaluate the impact of MedSafer deprescribing reports on 30-day postdischarge ADEs. The secondary objectives were to assess the impact of MedSafer reports on deprescribing and on safety outcomes related to adverse drug withdrawal events (ADWEs).

## Methods

We evaluated the use of MedSafer using a cluster randomized trial at 11 participating acute care hospitals in Canada. All hospital sites obtained study approvals from their local research ethics board and administration. Potentially eligible patients were approached for their consent to the 30-day postdischarge telephone interview; a family member or proxy granted consent for patients lacking capacity, in accordance with the ethics regulations of Canada.^18^ The trial protocol and statistical analysis plan are available in Supplement 1. The study followed Consolidated Standards of Reporting Trials (CONSORT) reporting guidelines extension for cluster randomized trials.^19^

### Design, Study Population, Setting, and Randomization

The study was conducted with patients admitted to a participating site from August 22, 2017, to January 13, 2020. Each participating site had a control phase followed by an intervention phase. The timing of the intervention varied by cluster, and the order of entry into the intervention was determined centrally by a randomized sequence generated by statistical software. Study sites were kept blinded until approximately 4 weeks prior to their allocation to the intervention to allow for preparation. Clusters were scheduled to move from 1 period to the next every approximately 500 recruited patients. One cluster (Western Canada) had 2 periods when they were able to recruit only 250 participants of the planned 500 because patients had been transferred to nonstudy units.

Patients aged 65 years and older who were regularly taking 5 or more usual medications prior to admission were eligible. Patients receiving palliative care with an expected prognosis of more than 3 months were included. Readmitted patients were eligible only if they had not been previously enrolled in the study. Additional study design details, including the composition of the clusters, are available in the eMethods, eFigure 1, and eTables 1 and 2 of Supplement 2.

### Description of the Intervention

During the control period, patients received usual care (having a best-possible medication history performed), with medication reconciliation at hospital admission and discharge.^20^ Any deprescribing that took place was based on local and individual practice by the unit physicians and pharmacists. A trained research assistant extracted medical conditions and specific laboratory values from the comprehensive admission notes and entered these data, along with the prehospital medications list, into MedSafer. Any changes to home medications at discharge were captured from exit prescriptions and/or medication reconciliation documents.

The main intervention was the provision of individualized deprescribing reports based on evidence-based guidelines for safer prescribing in older adults,^21,22,23^ with tapering instructions when indicated. The report prioritized opportunities for deprescribing based on prespecified expert consensus as either: (1) high-risk PIMs (harms outweigh benefits for most); (2) intermediate-risk PIMs (harms may approximate benefits, clinical judgment required); and (3) PIMs of little added value (medication shown to be ineffective or adds to pill burden). Opportunities for deprescribing were generated by cross-referencing medical conditions, laboratory values, and home medication lists with evidence-based guidelines for safer prescribing in older adults, described previously.^16,24^

Deprescribing reports were provided to the treating team within 3 business days of patient admission and were designed to engage the physician and the hospital pharmacy team. At discharge, the report was sent by facsimile to the patient’s community pharmacy and the self-identified usual treating physician(s). Patients or their caregivers received an educational pamphlet on deprescribing, as well as relevant patient-oriented deprescribing pamphlets from the Canadian Deprescribing Network for select classes of medications, such as sedative-hypnotics.^25^ Other site-specific procedures are detailed in the eMethods of Supplement 2.

Patients were assessed according to the Clinical Frailty Scale.^26^ If capable of doing so, patients completed the PROMIS SD 4a (Patient-Reported Outcomes Measurement Information System short form, version 1.0, sleep disturbance 4a^27^) to describe their sleep status before and after hospitalization.

### Outcomes

The primary outcome was the proportion of patients experiencing an ADE within the first 30 days after hospital discharge in the intervention vs the control phases, including ADWEs, which are a subset of ADEs (eFigure 2 in Supplement 2). Patients or proxies consented to a structured telephone interview approximately 30 days after hospital discharge, conducted by an experienced interviewer and blinded to the intervention status.^28^ The interview consisted of a modified Australian Adverse Reaction and Drug Event report^29^ and questions about medication changes, new or worsening symptoms, and planned or unplanned visits to medical professionals. Details of the interview were previously described^16^ and are available in the eMethods of Supplement 2. If a patient was readmitted to the hospital, a detailed review of the EHR was performed and the reason(s) for readmission was identified.

The adjudication of ADEs was performed by trained study investigators blinded to patient identification, hospital site, province, and the intervention status. In brief, reviewers were first asked to determine if an adverse event had occurred (eg, a fall, emergency department visit, hospitalization, unplanned health care visit) and then, using a Leape and Bates Likert scale,^30^ to rate the likelihood of it having been an ADE. An ADE was defined as an event with a rating of 5 (probably caused by medication) or 6 (definitely caused by medication).^30,31^ Adjudication was conducted independently in duplicate, and disagreements were resolved by an independent third reviewer. Details of training and examples are available in the eMethods of Supplement 2 and also have been described elsewhere.^31^ The Gwet agreement coefficient (Gwet AC)^32^ was used to estimate interobserver agreement between the first 2 reviewers for adverse events and ADEs, respectively. Safety outcomes included ADWEs, whereby the reviewer was asked if the ADE was owing to temporarily withholding, tapering, or stopping (deliberately or by omission) of a medication. A sensitivity analysis was performed that included any ADE that was rated as a 4 (possibly related to medication) or higher on the Leape and Bates Likert scale.

Secondary outcomes included the proportion of patients with 1 or more PIMs deprescribed at hospital discharge compared with usual care. A medication was considered deprescribed if it was discontinued or if a taper to discontinue was prescribed. For medications whose dose was reduced, a blinded review was used to determine if that reduction was a deliberate attempt at deprescribing. We also evaluated 30-day postdischarge adverse effects, falls, emergency department visits and/or hospitalizations, deaths, quality of life (using the EQ-5D-5L questionnaire with a Canadian value set^33^), and sleep disturbance (using the PROMIS SD 4a). Harms were considered ADWEs, as were the impacts on sleep and quality of life. Prespecified subgroup analyses examined the effect by sex, palliative designation, frailty, and residence in a long-term care facility.

### Sample Size

Based on the literature, the proportion of medical patients who could experience an ADE after discharge was estimated to be 15.0%.^34,35^ We used an α of .05, 80% power, and an expected cluster correlation coefficient of 0.03.^36^ We prespecified an absolute decrease of approximately 4.0% corresponding to a number needed to treat of 25. Using the Stata stepped-wedge function^37^ and following the Hussey and Hughes approach,^38^ we planned for a 6-cluster trial with each cluster recruiting 200 patients per cluster-period for a total of 8400 patients, resulting in 80% power to detect a change in ADE from 15.0% to 11.2%.

Prior to the recruitment of 200 participants in any cluster, recruitment rates suggested it would be impossible to complete the trial as originally designed. Without examining outcomes data, we realigned the 6 clusters into 3 clusters according to recruitment rates and geography (Ontario, Quebec, and Western Canada), permitting the study to be completed within the funding duration and budget. A 3-cluster trial with 500 patients per cluster per period allowed for 80% power to detect a 4.8% absolute difference with 6000 patients.

### Statistical Analysis

Baseline characteristics were expressed as numbers and percentages for categorical variables and median (IQR) for continuous variables. Differences between control and intervention baseline characteristics were compared by χ^2^ or rank-sum as appropriate.

For the primary outcome, ADEs, a mixed-effects logistic regression model was used controlling for intervention status, time period, and the number of baseline PIMs as fixed effects, and cluster as a random effect. Adjusted risk differences (aRD) were estimated from the model parameters differences.^39^ This analysis was conducted with data from patients who consented and participated in the postdischarge follow-up interview.

For secondary outcomes, an identical analysis was conducted for any postdischarge adverse event. The intervention’s effectiveness in terms of stopping 1 or more PIM was also evaluated using the same method, restricted to those participants taking 1 or more PIM at admission and discharged alive. Further details of the analysis are described in the eMethods of Supplement 2.

### Sensitivity Analyses

To address the low number of clusters in the study, we performed 5 post hoc sensitivity analyses: (1) an analysis adjusting for patient factors that differed between groups by *P* < .01; (2) treating clusters as a fixed effect^40^; an analysis with (3) a random and (4) fixed effect for hospital (n = 11, in lieu of cluster), and (5) using permutation, a nonparametric method of evaluating for the weighted within period effect size.^41,42^ All statistical tests were 2-tailed and *P* values < .05 were considered statistically significant. Data analyses were performed from January 3, 2021, to September 23, 2021, using Stata, version 16 (StataCorp LLC).

## Results

A total of 11 922 older patients (≥65 years) taking 5 or more medications were admitted to 1 of the study sites from August 22, 2017, to January 13, 2020; of these, 6633 were eligible for and enrolled in the study. The study analyses included the 5698 patients (median [IQR] age, 78 [72-85] years; 2858 [50.2%] women; race and ethnicity data were not collected) who survived to hospital discharge (control, 3204 patients; intervention, 2494 patients; Table 1). Of these, 4989 patients (87.5%) completed a postdischarge interview (control, 2742 patients; intervention, 2247 patients; Figure). Deprescribing opportunities were identified in 4923 patients (86.4%).

**Table 1.** Patient Characteristics

Characteristic	No. (%)	
	Control (n = 3204)	Intervention (n = 2494)
Demographic information		
Age, median (IQR)	78 (71-85)	78 (72-86)
Female sex	1619 (50.5)	1239 (49.7)
Primary spoken language, English	2859 (89.2)	1782 (71.5)
Admitted from long-term care facility	165 (5.1)	185 (7.4)
Medications		
No. of home medications, median (IQR)	10 (8-13)	10 (8-14)
No. of PIMs identified, median (IQR)	2 (1-3)	2 (1-4)
Length of stay, median (IQR)	7 (4-13)	8 (5-15)
Comorbidity		
HOMR score, median (IQR)	39 (36-41)	39 (36-41)
Hypertension	2348 (73.3)	1875 (75.2)
Congestive heart failure	999 (31.2)	803 (32.2)
Valvular heart disease	623 (19.4)	544 (21.8)
Ischemic heart disease	1085 (33.9)	868 (34.8)
Atrial fibrillation	1035 (32.3)	794 (31.8)
CHADS2, median (IQR)	3 (2-3)	3 (2-3)
Ischemic stroke (ever)	388 (12.1)	343 (13.8)
Venous thromboembolism (ever)	276 (8.6)	217 (8.7)
Gastrointestinal hemorrhage	358 (11.2)	237 (9.5)
Peptic ulcer disease	147 (4.6)	111 (4.5)
Gastroesophageal reflux disease	688 (21.5)	499 (20.0)
Cirrhosis	142 (4.4)	112 (4.5)
Diabetes (type 2)	1272 (39.7)	973 (39.0)
Glycated hemoglobin A_1c_, median (IQR)^a^	7.1 (6.3-8.2)	7.0 (6.3-8.2)
History of chronic kidney disease	802 (25.0)	728 (29.2)
Chronic obstructive pulmonary disease	832 (26.0)	614 (24.6)
Solid organ cancer	814 (25.4)	683 (27.4)
Generalized anxiety or major depression	554 (17.3)	422 (16.9)
Major neurocognitive disorder	396 (12.4)	438 (17.6)
Delirium (ever)	464 (14.5)	412 (16.5)
Recurrent falls	542 (16.9)	636 (25.5)
Clinical frailty scale, median (IQR)^b^	4 (3-5)	4 (4-5)

Abbreviations: CHADS2, congestive heart failure, hypertension, age, diabetes, and stroke; HOMR, hospital patient 1-year mortality risk; PIM, potentially inappropriate medication.

^a^ Data for 879 and 709 patients, respectively.

^b^ Data for 3150 and 2453 patients, respectively.

**Figure.**
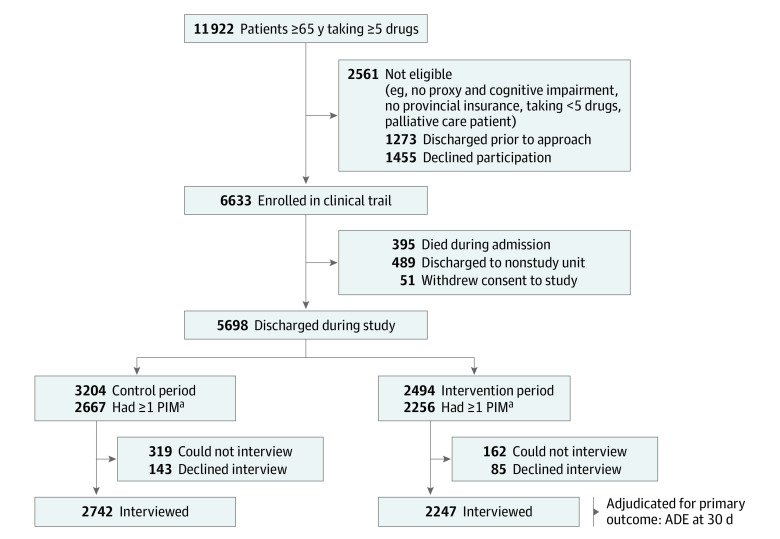
CONSORT Flow Diagram of Older Patients Admitted to and Discharged From 11 Hospitals in Canada ^a^Adjudicated for the secondary outcome, ie, deprescribing effectiveness at discharge. ADE denotes adverse drug event; PIM, potentially inappropriate medication.

### Baseline Data

The control and intervention groups were similar regarding most major medical comorbidities; however, the intervention group was slightly older and more likely to have a diagnosis of a major neurocognitive disorder. The median (IQR) number of home medications was 10 (8-14); median (IQR) number of PIMs was 2 (1-3); and the median (IQR) hospital patient 1-year mortality risk (HOMR) score was 39 (36-41) or approximately a 30% risk of death within 1 year.^17^ The most frequently identified PIMs were proton pump inhibitors, sedative hypnotics, gabapentinoids, and diabetic agents with low (<7.5%) glycated hemoglobin A_1c_ levels.

### Primary Outcome

Among the control participants, 138 (5.0%) of 2742 had an ADE vs 111 (4.9%) of 2247 of intervention patients (aRD, −0.8%; 95% CI, −2.9% to 1.4%; Table 2). The most reported ADE was bleeding and anemia, followed by fluid overload (often classified as an ADWE), followed by acute kidney injury and electrolyte disorders and falls (eTable 3 in Supplement 2). Interobserver reliability was excellent for the classification of ADEs (Gwet AC, 0.92) and adverse events (Gwet AC, 0.81). Common culprit medications included direct oral anticoagulants, diuretics, opioids, and prednisone. The sensitivity analysis that included possible ADEs (≥4 on the Leape and Bates Likert scale) found an overall aRD of −2.3% (95% CI, −4.9% to 0.4%) in favor of the intervention (eTable 4 and eFigure 3 in Supplement 2).

**Table 2.** Adverse Drug Events by Cohort, Period, and Intervention Status

Cohort	No. (%)					
	Period 1	Period 2	Period 3	Period 4	Total control	Total intervention
Cohort 1						
No. of patients	426	467	465	508	1358	508
ADE	19 (4.5)	18 (3.9)	24 (5.2)	22 (4.3)	61 (4.5)	22 (4.3)
Any adverse event	125 (29.3)	136 (29.1)	153 (32.9)	159 (31.3)	414 (30.5)	159 (31.3)
Cohort 2						
No. of patients	415	427	245	247	842	492
ADE	28 (6.8)	27 (6.3)	11 (4.5)	16 (6.5)	55 (6.5)	27 (5.5)
Any adverse event	147 (35.4)	134 (31.4)	65 (26.5)	80 (32.4)	281 (33.4)	145 (29.5)
Cohort 3						
No. of patients	542	408	428	411	542	1247
ADE	22 (4.1)	20 (4.9)	18 (4.2)	24 (5.8)	22 (4.1)	62 (5.0)
Any adverse event	184 (34.0)	130 (31.9)	136 (31.8)	114 (27.7)	184 (34.0)	380 (30.5)
Intervention						
No. of patients	NA	408	673	1166	NA	2247
ADE	NA	20 (4.9)	29 (4.3)	62 (5.3)	NA	111 (4.9)
Any adverse event	NA	130 (31.9)	201 (29.9)	353 (30.3)	NA	684 (30.4)
Before intervention						
No. of patients	1383	894	465	NA	2742	NA
ADE	69 (5.0)	45 (5.0)	24 (5.2)	NA	138 (5.0)	NA
Any adverse event	456 (33.0)	270 (30.2)	153 (32.9)	NA	879 (32.1)	NA

Abbreviations: ADE, adverse drug events; NA, not applicable.

### Secondary Analyses

The number of intervention participants with 1 or more PIMs deprescribed increased substantially, from 795 (29.8%) to 1249 (55.4%; aRD, 22.2%; 95% CI, 16.9% to 27.4%; Tables 3 and 4); 92.8% of the deprescribed medications remained stopped at 30 days postdischarge (vs 89.4% in the control). In the control group, 879 (32.1%) participants had an adverse event within 30 days compared with 684 (30.4%) in the intervention (aRD, −1.2%; 95% CI, −6.4% to 4.1%; Table 2). The incidence of postdischarge falls decreased insignificantly (odds ratio, 0.76; 95% CI, 0.57 to 1.05). Additional secondary outcomes were similar between groups; results of prespecified subgroup analyses are presented in eTables 5 to 10 and eFigures 4 and 5 in Supplement 2.

**Table 3.** Proportion of Patients With 1 or More PIM Deprescribed (Stopped) by Cohort, Period, and Intervention Status

Cohort	No. (%)					
	Period 1	Period 2	Period 3	Period 4	Total control	Total intervention
Cohort 1						
No. of patients	429	457	447	513	1333	513
≥1 PIM stopped	114 (26.6)	80 (17.5)	87 (19.5)	204 (39.8)	281 (21.1)	204 (39.8)
Cohort 2						
No. of patients	409	419	257	256	828	513
≥1 PIM stopped	161 (39.4)	125 (29.8)	162 (63.0)	131 (51.2)	286 (34.5)	293 (57.1)
Cohort 3						
No. of patients	506	410	422	398	506	1230
≥1 PIM stopped	228 (45.1)	230 (56.1)	265 (62.8)	257 (64.6)	228 (45.1)	752 (61.1)
Intervention						
No. of patients	NA	410	679	1167	NA	2256
≥1 PIM stopped	NA	230 (56.1)	427 (62.9)	592 (50.7)	NA	1249 (55.4)
Before intervention						
No. of patients	1344	876	447	NA	2667	NA
≥1 PIM stopped	503 (37.4)	205 (23.4)	87 (19.5)	NA	795 (29.8)	NA

Abbreviations: PIM, potentially inappropriate medications; NA, not applicable.

**Table 4.** Deprescribing Rates of Commonly Alerted Potentially Inappropriate Medications

Specific PIMs	Possible problem	Control (n = 2667)			Intervention (n = 2256)			% Difference (95% CI)	
		Users (%)	PIM (%)	PIM deprescribed (%)	Users (%)	PIM (%)	PIM deprescribed (%)	Unadjusted	Adjusted
Benzodiazepines and sedative hypnotics^a^	Increased risk of delirium, falls, death	665 (24.9)	553 (83.2)	113 (20.4)	538 (23.8)	524 (97.4)	210 (40.1)	19.6 (14.3 to 25.0)	22.7 (12.0 to 33.5)
Codeine and tramadol^b^	Unpredictably metabolized. If opioids are needed, a safer choice should be made	272 (10.2)	216 (79.4)	74 (34.3)	182 (8.1)	179 (98.4)	98 (54.7)	20.5 (10.8 to 30.1)	43.0 (30.5 to 55.5)
Combination antiplatelet and anticoagulants	Increased risk of bleeding; may be inappropriate	269 (10.1)	215 (79.9)	75 (34.9)	173 (7.7)	146 (84.4)	65 (44.5)	9.6 (−0.6 to 19.9)	24.8 (8.0 to 41.7)
Opioids (excluding codeine and tramadol) ^b^	Opioid use outside of cancer pain is associated with risk of death	430 (16.1)	201 (46.7)	57 (28.4)	374 (16.6)	210 (56.1)	83 (39.5)	11.2 (2.1 to 20.3)	17.8 (−2.4 to 37.9)
Trazodone^a^	Off-label use for sleep is not indicated	231 (8.7)	156 (67.5)	23 (14.7)	132 (5.9)	92 (69.7)	30 (32.6)	17.9 (6.8 to 28.9)	24.3 (2.2 to 46.5)
Nonsteroidal anti-inflammatories	Can exacerbate congestive heart failure or hypertension	230 (8.6)	155 (67.4)	36 (23.2)	145 (6.4)	120 (82.8)	42 (35.0)	11.8 (1.0 to 22.6)	12.7 (−3.2 to 28.7)
Antipsychotics^a^	Not recommended as first line treatment for sleep or agitation in dementia	239 (9.0)	144 (60.3)	33 (22.9)	238 (10.5)	206 (86.6)	70 (34.0)	11.1 (1.6 to 20.5)	12.9 (−6.2 to 32.1)
Mirtazapine^a^	Off-label use for sleep is not indicated	136 (5.1)	54 (39.7)	5 (9.3)	122 (5.4)	62 (50.8)	12 (19.4)	10.1 (−2.4 to 22.6)	4.4 (−11.2 to 20.0)
Proton-pump inhibitors	Frequently used without indication	1442 (54.1)	1227 (85.1)	127 (10.4)	1149 (50.9)	1056 (91.9)	222 (21.0)	10.7 (7.7 to 13.7)	9.4 (2.5 to 16.4)
Diabetes therapies^c^	Demonstrated hypoglycemia; contraindicated agents in kidney failure	948 (35.5)	436 (46.0)	159 (36.5)	756 (33.5)	381 (50.4)	192 (50.4)	13.9 (7.2 to 20.7)	11.3 (−2.3 to 25.0)
Gabapentinoids	Frequently used off label and have many adverse effects (fluid retention, worsening cognition, and death)	558 (20.9)	406 (72.8)	86 (21.2)	367 (16.3)	323 (88.0)	114 (35.3)	14.1 (7.6 to 20.7)	0.6 (−11.6 to 12.9)
Thiazides	High risk of hyponatremia if prior hyponatremic event	467 (17.5)	152 (32.5)	78 (51.3)	356 (15.8)	129 (36.2)	101 (78.3)	27.0 (16.3 to 37.6)	32.8 (17.4 to 48.2)
SSRIs	Can contribute to recurrent falls in older adults	407 (15.3)	91 (22.4)	16 (17.6)	351 (15.6)	88 (25.1)	19 (21.6)	4.0 (−7.6 to 15.6)	14.8 (−4.6 to 34.1)
High-dose iron salts^d^	Less tolerated and no more effective than standard dosage	535 (20.1)	129 (24.1)	21 (16.3)	398 (17.6)	109 (27.4)	54 (49.5)	33.3 (21.9 to 44.6)	26.5 (1.2 to 51.7)
Docusate	Ineffective for treatment or prevention of constipation	248 (9.3)	248 (100.0)	99 (39.9)	208 (9.2)	208 (100.0)	133 (63.9)	24.0 (15.1 to 33.0)	23.4 (5.6 to 41.2)
Nonstatin cholesterol medications^c^	Limited evidence of efficacy	145 (5.4)	137 (94.5)	12 (8.8)	120 (5.3)	120 (100.0)	35 (29.2)	20.4 (11.0 to 29.8)	12.7 (−8.3 to 33.8)

Abbreviations: PIM, potentially inappropriate medication; SSRI, selective serotonin reuptake inhibitors.

^a^ Excludes patients with psychiatric indication (or seizure for benzodiazepines).

^b^ Excludes patients in palliative care or with cancer as possible indication.

^c^ Users may have been taking >1 medication, and user numbers represent ≥1.

^d^ Excludes those already taking low-dose iron salts.

### Harms

There were 49 ADWEs representing 19.7% of 249 ADEs (aRD, −0.1%; 95% CI, −1.2% to 1.0%). The most common ADWE was fluid overload secondary to dose adjustment of diuretics, with 1 event owed to a MedSafer recommendation—hydrochlorothiazide deprescription triggered by severe hyponatremia. Sleep and quality of life remained stable before and after hospitalization (eTables 5 to 10 in Supplement 2).

### Sensitivity Analyses

All 5 sensitivity analyses provided similar estimates of effect size and confidence intervals. None of the sensitivity analyses altered the conclusions of the trial. These results are presented in eTable 11 of Supplement 2.

## Discussion

Despite clinically and statistically significant increases in deprescribing, communication of medication changes to community physicians and pharmacists, and sustained deprescribing 30 days postdischarge, this large randomized clinical trial was unable to demonstrate any significant impact of deprescribing decision support on short-term ADEs. Several explanations may address the discrepancy between the impact on deprescribing and the complete lack of effect on ADEs. The overall incidence of ADEs (defined as a Leape and Bates Likert score of 5 or 6) was only 5%, which is lower than the approximately 10% to 15% incidence observed in the seminal studies^2,34,35^ used to inform our power calculations. However, those studies were conducted more than 15 years ago. The complexities of patient and medication regimens have increased substantially and identification of probable and/or definite ADEs has become more challenging.^31^ In addition, widespread hospital pharmacist involvement in medication reconciliation has created opportunities to mitigate more worrisome prescribing practices, such as errors of omission, which may lead to clearly identifiable ADEs.

While our intervention identified numerous deprescribing opportunities, many were for low-risk nonbeneficial polypharmacy (eg, nonstatin cholesterol-lowering medications or stool softeners^43^). Deprescribing these medications is less likely to impact 30-day ADEs, but still has patient and societal value, eg, avoiding excess cost, waste, and pill burden.^9,43^ When powering future studies of ADEs, interventions may need to focus specifically on high-risk medications, and the time frame for observing the outcome likely needs to be extended.^44^

To our knowledge, few large randomized clinical trials of deprescribing in acute care have been completed. The 1537 patients studied by SENATOR^15^ (Software Engine for the Assessment and Optimization of Drug and Nondrug Therapy in Older Persons Trial) found poor uptake (<20%) of computerized pharmacologic recommendations in their hospital setting. An accompanying qualitative study^5^ attributed this to multiple concurrent work commitments and a reluctance to take sole responsibility for an older person’s pharmacotherapy. Most other deprescribing studies have been small, potentially leading to questions of whether the absent impact on ADEs was related to study size. From our study findings, it appears that the timing of outcome ascertainment and the approach to quantifying medication harms are equally important, and both may need to be adapted for future studies.^31^

### Limitations and Strengths

This study had limitations; first among them was our low number of clusters (n = 3). When we reduced the number of clusters in the study, we introduced a risk that randomization might not achieve balance between groups across entry times, and indeed this was the case. For instance, the intervention group was slightly older, and participants had been prescribed more baseline PIMs. To attempt to address any bias or risk to validity owing to these imbalances, we performed several sensitivity analyses. Although these supported the conclusion of the study, the results still need to be interpreted with this caveat in mind.

Second, the rate of ADEs was significantly lower than expected, leaving the study underpowered for the primary outcome despite being the largest deprescribing trial performed to date to our knowledge. A recent study evaluated a multifaceted pharmacist-led intervention on medication safety posthospital discharge.^45^ The primary outcome included potential ADEs, in addition to preventable and ameliorable ADEs; however, it failed to demonstrate an impact on ADEs even with this broader definition. While our sensitivity analysis that included possible ADEs appeared to show a stronger effect of the intervention, it still did not demonstrate a reduction in ADEs.

Third, the generation of deprescribing reports required manual data input, which could limit scalability. The software is now updated to process EHR data (eg, codes from the *International Statistical Classification of Diseases and Related Health Problems, Tenth Revision*, and Drug Identification Numbers) directly via an application programming interface. This capability has important implications for implementation by reducing the burden of data input.

Fourth, reports were provided for home medications at hospital admission and not regenerated at discharge. Thus, the intervention did not formally address new in-hospital PIM starts,^44^ which may be particularly relevant to preventing early postdischarge ADEs.

Importantly, the intervention did not lead to an increase in ADWEs. The findings demonstrated a clinically and statistically significant impact on deprescribing of PIMs and generated extensive safety data in support of acute care deprescribing. Other strengths of the study included an intervention that addressed complex medication regimens and is generalizable to academic and community hospitals. We involved in-hospital pharmacists in the design of the intervention, which may have increased the proportion of deprescribing opportunities that were accepted. We also engaged patients and their caregivers with deprescribing brochures, and communicated medication changes to the treating community team, which promoted shared decision-making and reinforced longer-term persistence of changes made during the hospitalization.

## Conclusions

This randomized clinical trial found that providing deprescribing decision support to the acute care medical teams did not impact 30-day ADEs; however, this intervention effectively stopped PIMs, with no evidence of increased harm. Short-term ADEs may not be the ideal outcome to measure to capture the benefits of deprescribing during an acute care hospitalization.

## Notes

Supplement 1.
Trial Protocol

Supplement 2.
eMethods. Table of Contents and Section A-I
**eTable 1. **Details of the Timing of Deprescribing Report Reviews **eTable 2.** Additional Details of Academic Detailing by Site **eFigure 1.** Study Design Diagram **eFigure 2.** ADWEs are a subcategory of all ADEs **eTable 3.** Classification of Adverse Drug Events by Intervention Status **eTable 4.** Sensitivity Analysis for the Primary Outcome of 30-day Adverse Drug Events that Includes Grade 4 Possible Adverse Drug Events (ADE) **eFigure 3. **Subgroup effects by Intervention Status for the Primary Outcome of 30-day Adverse Drug Events **eTable 5.** PROMIS Sleep Disturbance Score Pre- and Post-hospitalization by Intervention Status **eTable 6.** Sleep Quality Pre- and Post-hospitalization by Intervention Status **eTable 7.** Sleep is Refreshing Pre- and Post-hospitalization by Intervention Status **eTable 8. **Trouble Sleeping Pre- and Post-hospitalization by Intervention Status **eTable 9.** Difficulty Falling Asleep Pre- and Post-hospitalization by Intervention Status **eTable 10.** Quality of Life by Intervention Status **eFigure 4. **Subgroup Effects by Intervention Status for the Secondary Outcome of Proportion with 1 or more Potentially Inappropriate Medications Deprescribed **eFigure 5.** Unadjusted Number of PIMs Stopped in Control vs Intervention **eTable 11.** Sensitivity Analyses

Supplement 3.
Data Sharing Statement
