# Supplementary material for: The MedSafer Study—Electronic Decision Support for Deprescribing in Hospitalized Older Adults: A Cluster Randomized Clinical Trial
Source: JAMA Intern Med. 2022 Jan 18;182(3):1–10. doi: 10.1001/jamainternmed.2021.7429 (PMC8767487; doi:10.1001/jamainternmed.2021.7429)

Section X: The MedSafer Protocol and Statistical Analysis Plan

The MedSafer Study: A Cluster Randomized Trial of Electronic Decision Support for Deprescribing in Acute Care

Research Protocol

Funding: Canadian Institutes of Health Research/Investigator

Sponsor:

Research Institute of the McGill University Health Centre
2155 Guy Street
Montreal, QC H3H 2R9

Principal Investigators:
Todd C. Lee MD MPH
and
Emily G. McDonald MD MSc

McGill University Health Centre
Centre for Outcomes Research and Evaluation
5252 De Maisonneuve boulevard
Montréal, QC H4A 3S9
514-934-1934x53333

June 5, 2017
Last update: October 25th, 2021

Table of Contents

Section 1: Key Roles…………………………………………………………….… p. 3
Section 2: Background Information……………………………………….…..... p. 4
Section 3: Trial Objectives and Purpose………………..…………………… p. 9
Section 4: Trial Design……………………………………………………………. p. 10
Section 5: Selection and Withdrawal of Subjects……………………………..... p. 15
Section 6: Treatment of Subjects…………………………………………….... p. 17
Section 7: Assessment of Efficacy……………………………………………… p. 18
- **Adjudication Manual…………………………………………………… p. 20**
- **Examples of Adverse Events and Adverse Drug Events……………. p. 28**

Section 8: Assessment of Safety………………………………………………. p. 31
Section 9: Statistics/Statistical Analysis Plan…………………………………. p. 32
Section 10: Direct Access to Source Data/Documents…………….………… p. 36
Section 11: Quality Control/Quality Assurance………………....……………. p. 36
Section 12: Ethics…………………………………………………………….…… p. 36
Section 13: Data Handling/Record Keeping………………………………..…. p. 38
Section 14: Financing and Assurance……………………………………....…. p. 38
Section 15: Publication Policy……………………………………………….…. p. 39
Section 16: Making Data Available for Secondary Use………………………. p. 39
Section 17: Protocol References…………………………………………….…. p. 40
Section 18: Screenshots of the MedSafer application……………………….. p. 43

Section 1- Key Roles:

McGill University Health Centre

Nominated Principal Investigator: Todd C. Lee MD MPH
Co-Principal Investigator: Emily G. McDonald MD MSc
Co-investigators: Andre Bonnici, Louise Papillon Ferland, Louise Pilote, Robyn Tamblyn, Louise Papillon-Ferland
Project Coordinator: Lina Petrella
Research Assistants: Sarah Elsayed, Kristen Moran, Yejim Kim, Manoja Chandralingam, Liliane Mefanche
Database Manager: Alek Lefebvre

University Health Network

Co-investigators: James Downar, Pete Wu, Rodrigo Cavalcanti, Tom MacMillan
Research Assistants: Matt Riley, Sarah Meilach, Ellen Koo
Pharmacy: Kiran Battu, Sandra Porter, Rachel Whitty

The University of Ottawa

Co-investigators: Allen Huang, Babak Rashidi
Project Coordinator: Allison Jennings
Research Assistants: Kristin Dorrance, Kira Slivitzky, David Yachnin
Pharmacy: Derek Dys

Kingston Health Sciences Centre

Co-investigators: Johanna Murphy
Research Assistants: Titania Dixon-Luinenburg

University of Calgary (Foothills)

Co-investigators: Gabriel Fabreau
Research Assistants: Kayla Atchison

University of Edmonton

Co-investigators: Finlay McAlister
Project Coordinator: Miriam Fradette
Research Assistants: Jennifer Shiu

University of British Columbia

Co-investigators: Anita Palepu, Nadia Khan
Project Coordinator: Anna Meredith
Research Assistants: Jasmine Lee

Section 2- Background information

2.1 Background

The prevalence of polypharmacy, or the concomitant use of five or more drugs, has dramatically increased since the 2000s, with at least half of elderly patients meeting this definition[1, 2]. With such high rates, excessive polypharmacy (the use of ten or more drugs) has now emerged in at least 20% of community-dwelling elderly and is commonly responsible for adverse drug events[3-5]. The frail elderly are at highest risk of excessive polypharmacy[5-7]. At McGill University there recently was a cluster-randomized controlled trial[8, 9], "RightRx" led by Dr. Robyn Tamblyn, examining electronic medication reconciliation software that links patient provincial drug insurance information to the in-hospital medication list. Recent (unpublished) analyses from the "RightRx" trial suggest that polypharmacy is exceedingly common amongst our elderly hospitalized patients, with more than 50% of those aged 65 and older discharged with excessive polypharmacy and 10% leaving the hospital with prescriptions for more than 20 usual daily medications. A by-product of polypharmacy is an increase in the number of drug-drug and drug-disease interactions with an increased potential for adverse drug events (ADEs)[9]. ADEs are common, with up to 35% of community-dwelling elderly having one each year[5, 6, 10]. Severe ADEs contribute in up to 20% of all hospitalizations in the elderly with an associated cost of nearly 36 million dollars per year in Canada[11].

There is a greater risk of ADEs when a patient is taking more medications and efforts to curtail polypharmacy could reduce this risk. However, countering polypharmacy is challenging, given patients with multiple medical conditions may legitimately require medications for each condition. Nonetheless, with time, a proportion of these medications eventually become unnecessary, inappropriate, or the harms outweigh potential benefits. At this point, deprescribing via the identification and discontinuation of unnecessary or potentially inappropriate medications (PIMs), may prove to be a solution to both polypharmacy and related ADEs[12]. Candidate medications for deprescribing can be identified using freely available, expert derived, peer reviewed lists including the: START/STOPP criteria, Beers Criteria, and Choosing Wisely Canada recommendations[13-15]. All told, these lists contain hundreds of medications and because of their complexity, it is difficult for physicians to know their entire contents by memory. When deprescribing, a patient's medications are actively reviewed with specific consideration given to stopping those with an unfavorable risk-benefit profile either alone or in combination with other drugs[12, 16, 17]. Despite the intuitive appeal of deprescribing, the development of an easy-to-use system that facilitates the process and that demonstrates concrete evidence of benefit and safety is required for physicians to adopt it into their everyday practice of preventive medicine.

A computer-based system could more quickly identify medications and cue the physician that deprescribing might prevent harm or prevent an adverse drug event. In this project, we have

updated our existing software, which identifies PIMs and generates instructions for the patient and physician for safe discontinuation. At the time of hospitalization, the patient's medications, comorbidities, and a measure of frailty will be entered into the software, which will generate an individualized and prioritized list of deprescribing opportunities to be considered as part of a deprescription plan.

2.2 Description of the intervention:

A) Implement an electronic deprescribing tool (*MedSafer*) to guide physicians and pharmacists through the process of safe and successful medication deprescription and determine the impact on 30-day post-hospital discharge adverse drug events.

B) Using anonymized data collected through chart review:

1) Describe the population of patients admitted to medical clinical teaching units (CTUs) or general medical wards in terms of anonymous demographics, medication profiles, and select aggregate in hospital outcomes (emergency department visits and hospitalizations, falls, and re-admissions post-discharge).

2) Determine the proportion of medications taken by patients, which would be deemed potentially inappropriate at admission and discharge based on expert derived lists.

3) Determine if explicitly informing doctors that their patients are receiving medications which evidence-based guidelines suggest may be inappropriate reduces the use of these medications by facilitating and automating the deprescribing process during the intervention period.

A trained research assistant will use the electronic deprescribing tool, *MedSafer*, to rapidly screen and cross-reference eligible patients' medical conditions (extracted from the internal medicine admission note or consult note) against their active community medication list derived from the best possible medication history. This tool can only be accessed by authorized research staff and is encrypted with password protection.

Screenshots of each page of the tool are included at the end of protocol.

The electronic deprescribing tool compares the patient's medication and past medical history data against a composite ruleset which has been adapted by the investigators based on their expertise and on existing published criteria for safer prescribing[13-15] and identifies those medications which are considered potentially inappropriate from the literature based on 1) risk of adverse drug event, or harm, relative to benefit or 2) lack of benefit or value.

In the control arm, all eligible participants will receive medication reconciliation at admission and discharge (current standard of care), and a structured follow-up phone interview to determine if they had an adverse event and adverse drug event within 30-days of hospital discharge. A prioritized deprescribing list will NOT be generated for patients during the control period of the study although the potentially inappropriate medications and any standard of care deprescribing decisions will be recorded.

During the intervention period, the medical teams will receive a printout with individualized and prioritized deprescribing opportunities: a) identifying the medication, b) explaining (with references and rationale) why that medication is potentially inappropriate and c) instructions on how to safely stop/taper the medication and what to look out for post discontinuation. The treating medical team will review these opportunities with the unit pharmacist and patient and subsequently appropriate candidate medications for deprescribing could be tapered or stopped by the most responsible physician on the CTU.

During the intervention period, for all patients who give consent for follow-up, the research assistants will provide a generic deprescribing brochure to inform them of the relevance of the study and the importance of medication review. Additionally, for those patients who are taking a proton pump inhibitor or a sedative hypnotic, they will receive corresponding patient-oriented educational material. The purpose of these brochures is to educate patients about the harmful effects of PPIs and sedative hypnotics. The patient brochures have been independently designed by the Canadian Deprescribing Network with input from patient stakeholders and the information is presented in lay terms in both French and English. Copies of each of the brochures are available online.

Proton pump inhibitors: http://www.criugm.qc.ca/fichier/pdf/PPI-EN-Men.pdf

Sedative hypnotics/sleeping pills: http://www.criugm.qc.ca/fichier/pdf/BENZOeng.pdf

2.3 Summary of findings from clinical studies that have potential implications:

Updated from the MedSafer pilot study: polypharmacy, or the concurrent use of multiple medications, is common, costly, and harmful[10, 11, 18]. As many as 40% of community-dwelling older adults are regularly prescribed five or more medications, despite being a population that is particularly vulnerable to adverse drug events (ADEs)[19-21]. Although several consensus-derived lists of potentially inappropriate medications (PIMs) for older adults exist that can be used as reference guides for comprehensive medication reviews, these lists can be challenging to translate into everyday practice. Lists of PIMs may not be applicable on an individual patient level and the process of cross-referencing multiple medications and medical conditions for the large number of patients who could benefit requires a command of the literature and is time consuming[22]. Previously described strategies to address polypharmacy have demonstrated

variable efficacy[23-27], with interventions led by pharmacists having the greatest success. Nevertheless, interventions can be limited by cost, time constraints, and the need for expert personnel who may be in short supply[28].

2.4 Summary of known potential risks and benefits:

Few studies have specifically looked for safety events related to deprescribing or "adverse drug withdrawal event" (ADWEs). Most studies of deprescribing have been underpowered to detect an impact on ADEs. This study will add important data to the generation of evidence in favour of deprescribing in acute care from a safety and efficacy point of view. Potential benefits include a reduction in pill burden, direct and indirect medication related costs, improved quality of life, and a reduction in adverse drug events. Potential harms include ADWEs (e.g., seizure after abrupt discontinuation of a benzodiazepine or gastrointestinal bleeding following cessation of a proton pump inhibitor).

2.5 Justification of the intervention:

Using the abstracted patient data, the web-based tool will identify medications which are (a) at high risk of an adverse drug event, (b) at intermediate risk of adverse drug event or for which clinical judgement is required or (c) are of expected to be of little added benefit or value. It will generate a prioritized and individualized list of potentially inappropriate medications and recommendations on how to safely discontinue or taper medications. These deprescribing opportunities are given to the patient's treating team for consideration (the CTU senior resident and unit pharmacist both receive a copy). This list is customized to the patient based on their medical history and the list of medications they are taking at admission. To avoid confusion or error on behalf of the treating teams (who have many patients under their care), the report is personalized with the patient's identifying information on the reports/recommendations. This will also allow for transmission of the medication changes after discharge to the patient's identified usual treating physician(s) and to their usual pharmacist(s) to avoid post-discharge medication errors from medication reconciliation.

The ruleset was derived from an expert consensus review of published or publicly available materials on safer drug use in older adults as well as from individual articles on specific medications and their risk of potential harm in the elderly[13-15].

Physicians (experts in geriatrics or those active in the field of "deprescribing") and pharmacists (particularly geriatric pharmacists) are already performing many of these acts implicitly. This is, in general, considered the standard of care in geriatric medicine. However, not all physicians/pharmacists who take care of older adults have a command of the literature, and the identification of potentially inappropriate medications can be complex when a given patient has numerous medical comorbidities and is taking multiple medications.

Competing priorities for healthcare providers restrict the time available for in-depth medication reviews and many teams across Canada do not have easy access to an expert in geriatric pharmacy. The purpose of our study is to provide support in terms of a rapid evidence-based and personalized screening of the patient's history and medications to contribute to safer prescribing in older patients.

It is our belief that the overall quality of prescribing on the units involved will increase, and our hope is that trainees will continue to practice safe prescribing through repeated exposure to the rulesets. This is one of the major goals/benefits of our project and why we were particularly interested in working on medical clinical teaching units across Canada.

2.6 Description of the population to be studied:

Consecutive adults aged 65 years and older on five or more usual home medications admitted to one of the study units at the participating hospitals. Participants are expected to have a prognosis of greater than three months, as judged by the treating team.

Section 3: Trial Objectives and Purpose (general statement)

Primary objective and secondary objectives: Deliver to the treating team a deprescribing opportunities report prior to the participant's hospital discharge to facilitate deprescribing. It is hypothesized that the intervention will lead to a reduction in Adverse Drug Events within 30 days post-discharge through the cessation of PIMs. Secondary outcomes will include adverse events at 30 days post hospital discharge as well as number of other measures listed subsequently.

Section 4: Trial Design

4.1 A specific statement of the primary endpoints and the secondary endpoints, if any, to be measured during the trial.

Primary outcome: Any adverse drug event within 30-days of hospital discharge (as defined by a 5 or 6 on the Leape and Bates scale[29]). ADEs could include adverse drug withdrawal events (ADWEs).

Secondary outcomes:

- Proportion of patients with one or more PIM discontinued at discharge
- Absolute number of medications at discharge
- Absolute number of PIMs at discharge
- Adverse drug withdrawal events within 30-days post discharge
- Absolute number of medications at 30 days post-discharge
    - Proportion of medications that remain deprescribed at 30 days post-discharge
- Adverse events within 30-days post discharge
    - 30-day readmissions (ER or hospitalization) within 30-days post discharge
    - 30-day mortality in those that survive to hospital discharge
    - Falls within 30-days of hospital discharge
- 30-day post-discharge sleep questionnaire responses
- 30-day self-reported health-related quality of life

Sensitivity analyses:

- Adverse drug events as defined by a 4,5 or 6 on the Leape and Bates scale

Effect by:

- Biological sex
- Admission from a long-term care home
- Palliative status
- Frailty status (dichotomized as moderate to severely frail vs not)

4.2 A description of the type/design of trial to be conducted (e.g., double-blind, placebo-controlled, parallel design) and a schematic diagram of trial design, procedures, and stages.

This is a cluster randomized trial comprising 3 clusters: Quebec, Ontario, and Western Canada (Alberta and British Columbia).

4.3 A description of the measures taken to minimize/avoid bias, including:

(a) Randomization

The order that the cluster enters intervention is randomized prior to the start of the study.

(b) Blinding and Allocation Concealment

Study sites will only learn that they are crossing over to the intervention 4 weeks prior.

Healthcare personnel and patients at participating sites will remain blinded to the specific objectives of the study while in the control phase. They will be aware that we are conducting a study of post-discharge adverse events; however, they will not know that we are specifically looking at medication appropriateness, deprescribing, and adverse drug events. When study sites cross into intervention it will no longer be possible to blind them to the objectives.

Adjudication of adverse events and adverse drug events is blinded and independent (the adjudicator does not know if the participant is in the intervention or the control phase of the trial). Adjudication only begins halfway through the second cluster entering intervention such that a significant proportion of participants are in intervention and control, thus minimizing the ability of the adjudicator to "guess" the intervention status.

Adjudication files are stripped of name of province, institution, hospital, doctors, and all specific dates (only relative dates are used). The patient file contains no identifiers and only a scrambled patient ID with randomly generated letters and numbers. Files from French hospitals and interviews will be fully translated to English prior to adjudication by personnel independent of the adjudication. Adjudications are randomly distributed to adjudicators using a computer-generated algorithm and are performed in duplicate. Where there is disagreement between reviews in terms of presence of an adverse event or adverse drug event, a 3rd independent blinded adjudicator will be randomly assigned. That adjudicator will not know they are performing a third review. We use agreement by 2 persons to constitute adverse drug events and adverse events.

4.4. The expected duration of subject participation, and a description of the sequence and duration of all trial periods, including follow-up, if any.

	Day 0	*Day 1-2*	*Day 2-3*	*Day 4*	*Day 5 to Day TBD*	*Day of D/C*	*Day of D/C +28- 35 Days*	*Day of D/C + >35 Days*
Hospital admission	*X*							
Medication reconciliation	*X*	*X*						
Patient consent		*X*	*X*					
Enter data for MedSafer report (medical history and medication list)		*X*	*X*					
Provide MedSafer report to team		*X*	*X*	*X*				
Team acts on recommendations		*X*	*X*	*X*	*X*	*X*		
Hospital discharge						*X*		
Discharge medication data collected						*X*		
30-day follow-up phone interview							*X*	
Adjudication of file								*X*

Study diagrams:

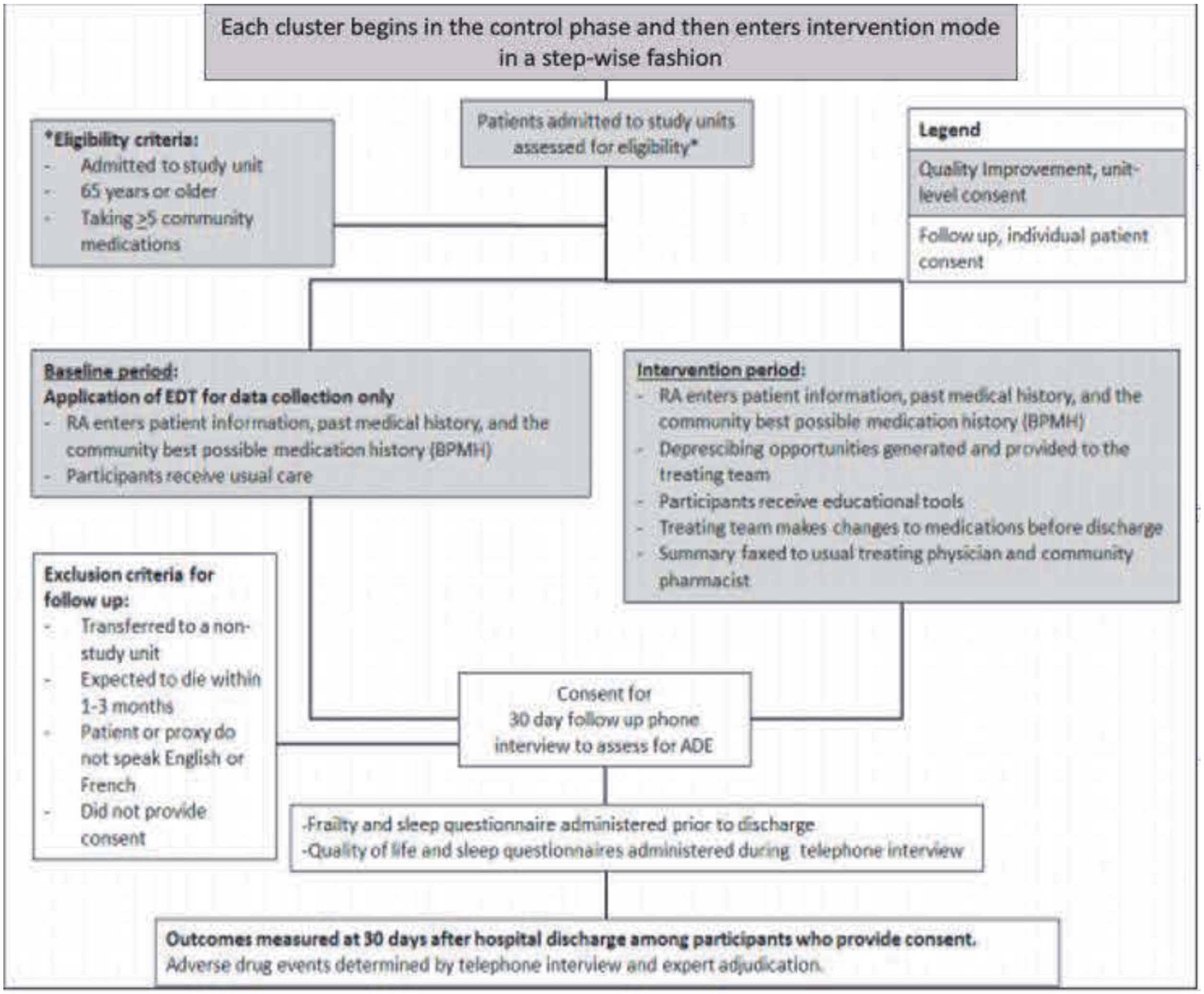

<u>**Study diagram updated from 6 clusters to 3 clusters (changed prior to first 200 patients)</u>

<u>Cluster randomized flow diagram: (updated to reflect 3 clusters instead of 6 prior to first 200 patients)</u>

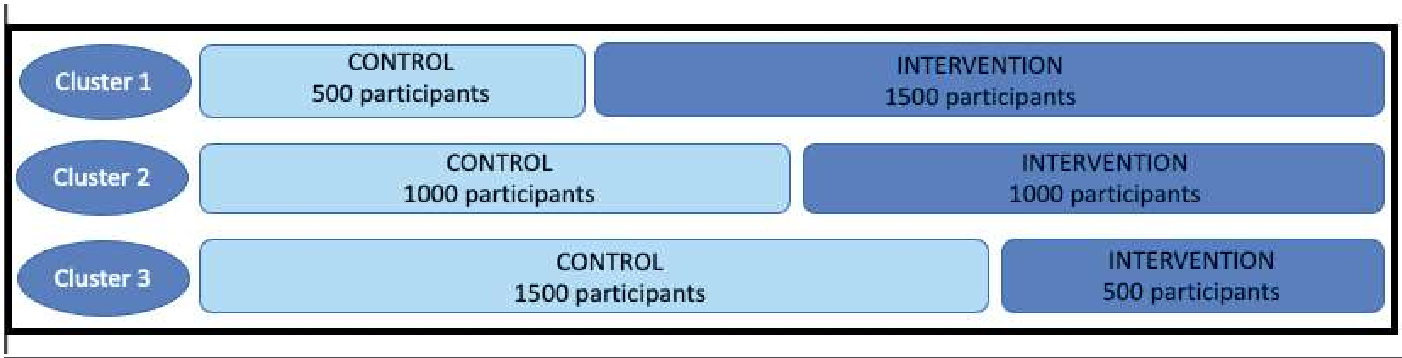

4.5 A description of the "stopping rules" or "discontinuation criteria" for individual subjects, parts of trial and entire trial.

Given the trial design (cluster-randomized) and to keep the case files blinded to intervention status, adjudication of the primary outcome will be delayed until sufficient time has passed with multiple groups in the intervention phase. Given the trial design and the need for duplicate reviews, it is not possible to have early stopping rules. The trial will complete enrolment. As the intervention is considered low risk and best practice, it is not expected that there will be serious adverse events linked to the intervention necessitating early stopping.

Section 5 – Selection and Withdrawal of Subjects

5.1 Subject identification and inclusion criteria.

5.1.a Specific inclusion criteria:

Participants eligible for this study include patients who are i) aged 65 years or older; ii) taking 5 or more medications; and iii) admitted to the study units.

5.1.b Subject exclusion criteria:

- Expected to die within 1-3 months
- Patient or proxy do not speak English or French
- Did not provide consent
- No provincial health insurance
- Will be unable to reach by telephone post-discharge
- Taking fewer than five usual home medications
- Admission or transfer to a non-study unit is expected: patients who are ultimately discharged from non-study units during their hospitalization will be excluded unless that unit is a transitional care, rehabilitation, or post-acute care unit used to bridge the gap between acute medical hospitalization and community services. For example, patients transferred to and subsequently discharged from a surgical unit (where there is no intervention) will be excluded.

5.2 When and how to withdraw subjects from the trial:

Withdrawal by participant: Participants will be able to withdraw from the study at any time upon request without penalty. Should participants discontinue, we will request that data accrued to date be retained for analysis but will terminate future follow up. We will also request permission to confirm ER visit, hospitalization, and vital status at day 30 post-hospital discharge.

Withdrawal by investigator: If any clinical adverse event (AE), laboratory abnormality, or other medical condition or situation occurs such that continued participation in the study would not be in the best interest of the participant or interferes with the integrity of the study data. If the participant meets an exclusion criterion (either newly developed or not previously recognized) that precludes further study participation.

5.3 The type and timing of the data to be collected for withdrawn subjects.

Vital status and hospitalization at 30-days post hospital discharge

5.4 Whether or how subjects are to be replaced:

Not applicable

5.5. The follow-up for subjects withdrawn:

If they agree, a 2-minute phone call at 30-days post hospital discharge to determine if they visited the emergency department or were rehospitalized and to determine vital status.

Section 6 – Treatment of Subjects

6.1 The intervention to be administered, including a description of the intervention, the timing, the method of administration, the follow-up period in the intervention and control arms

The intervention consists of an electronically generated deprescribing report. A trained research assistant will collect the past medical history and certain lab values from the electronic chart and the medical admission note. They will enter this information along with the best possible medication history. The medication history will be elicited by a pharmacist or pharmacy technician. The information will be entered into the software MedSafer and a report will be generated based on cross-referencing with rules based on the existing evidence-based guidelines for deprescribing[30].

The report will only be generated after the medication reconciliation is performed by the pharmacist. In general, the report will be provided as soon as possible to the treating team and could be on day 0 or admission and up to day 3 of admission depending on resources on the unit and weekday or weekend admission status.

The team is free to act on the reports as per their local practice. At their own discretion, teams may choose to review reports at sign out, during rounds with pharmacists or prior to discharge.

Reports could be acted upon (or not) prior to hospital discharge. The discharge prescription is used as the final determination of whether deprescribing reports are acted on.

Participants who are taking sleeping pills or PPIs will also receive a brochure from the Canadian Deprescribing Network detailing the rationale for deprescribing this medication. All participants will receive a one-page study brochure that explains the rationale behind deprescribing which is available at https://pamphlet.medsafer.org

Participants will be phoned within and around 30-days post discharge (day 28-35) and they will have a structured interview to determine their current medication regimen and if it has changed, along with any contact (planned or unplanned) with the healthcare system, and any new or worsening conditions[31]. They will also have the opportunity to report on their quality of sleep (Patient-Reported Outcomes Measurement Information System Sleep Disturbance Form 4a[32]) and quality of life (EQ-5D-5L)[33]. All information will be collected in an electronic case file report for later blinded adjudication by an expert in pharmacy, geriatrics, or general internal medicine.

At the time of consent, we will also request access to provincial health insurance data up to one-year post hospital discharge in jurisdictions where available for use in subsequent analyses.

6.2 Medication(s)/treatment(s) permitted (including rescue medication) and not permitted before and/or during the trial
Participants are not precluded from enrolling in other studies concurrently.

6.3 Procedures for monitoring subject compliance
Not applicable

Section 7 – Assessment of Efficacy

7.1 Specification of the efficacy parameters.

Primary outcome:
Any adverse drug event within 30-days of hospital discharge (as defined by a 5 or 6 on the Leape and Bates scale)[29].

Secondary outcomes(s):

- **Proportion of patients with one or more PIM discontinued at discharge**: determined from the discharge prescription as compared to the best possible medication history performed on admission (medication reconciliation). Medications which have been deliberately tapered to lower doses at discharge will be classified based on a blinded expert review as to whether they constitute "deprescribing".
- **Absolute number of medications at discharge:** determined from the discharge prescription.
- **Absolute number of PIMs at discharge**: once discharged the MedSafer algorithm will be run on the discharge medication list and PIM status recorded.
- **Adverse drug withdrawal events within 30-days post discharge:** this constitutes a subclass of adverse drug event whereby the adjudicator believes that the patient suffered an adverse event as the consequence of stopping, holding, omitting, decreasing or deprescribing a medication.
- **Absolute number of medications at 30 days post-discharge:** determined from post-discharge interview and review of medications.
    - **Proportion of medications that remain deprescribed at 30 days post-discharge:** determined by asking patients if they have restarted medications which were classified as being deprescribed at discharge.
- **Adverse events within 30-days post discharge:** determined by review of the post-discharge interview.
    - **30-day readmissions (ER or hospitalization) within 30-days post discharge:** a subset of adverse events related to emergency room visit or unplanned readmission to hospital. Details abstracted from the admission note and the death certificate/discharge summary.
    - **30-day mortality in those that survive to hospital discharge:** a subset of adverse events related to death (either in hospital or not in hospital). Where patients have died out of hospital, a modified interview of next of kin will be performed to obtain basic details about the death and this will be adjudicated. Where patients have been readmitted prior to death,
    - **Falls within 30-days of hospital discharge:** based on patient self report at the discharge interview.
- **30-day post-discharge sleep questionnaire responses:** as assessed from the patient interview using the questionnaire.
- **30-day self-reported health-related quality of life:** as assessed from the patient interview using the questionnaire.

Sensitivity analyses: Adverse drug events as defined by a 4,5 or 6 on the Leape and Bates scale

7.2 Methods and timing for assessing, recording, and analysing efficacy parameters.

Post-discharge interviews will be performed by trained research staff and will use a standardized questionnaire to obtain the information necessary to help determine if an adverse event has occurred and obtain an estimate of the patient's sleep quality and post-discharge health-related quality of life.

These post-discharge surveys will be combined with patient demographics into a de-identified record as soon as the telephone interview has been completed. Trained adjudicators will review these records and determine if an adverse (drug) event occurred post-discharge.

We will also, with consent, collect data on prescribed medications, hospitalizations and medical services that any eligible participant receives 12 months prior to their enrolment in the study, during their hospital admission at the time of enrolment, and up to 12 months after their hospital admission. Subsequently, when sufficient funding is available and/or provincial access pathways are clarified we will perform a pre-planned secondary analysis on those with long-term administrative data.

Patients will have the option of consenting for both the telephone interview and access to medication and hospitalization data for up to 1-year post-discharge, data access only, or interview only.

7.3 Methods for adjudicating adverse drug events:

Summary

We use a modification of the Leape and Bates method. First the adjudicator reviews the file and decides if any adverse event has happened. If not, the review is complete. If yes, the reviewer assigns a score based on the Leape and Bates scale with 5 and 6 representing ADE. The reviewer then identifies which events they believe represent the adverse event and, if ADE, which drugs they believe may or may not have been revised. If there is an ADE identified, the reviewer also rates whether it may have represented an adverse drug withdrawal event.

Each file is randomly assigned to 2 reviewers. In the event of a disagreement as to adverse event occurrence or adverse drug event occurrence, a third reviewer is randomly selected (and is blinded and independent to perform a third review).

Adverse drug events and adverse events are those which were categorized as such by 2 or more reviewers under the above process.

Adjudication Manual:

Log onto MedSafer trial application using Chrome-based browser/

1. Once logged on, select Reviewer role in the top left corner of the page.

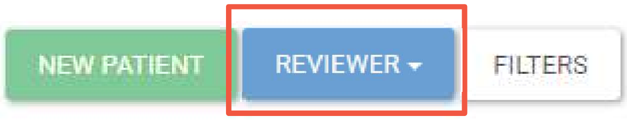

2. You will be able to see a list of patients assigned to you for adjudication. You can keep track of your patient if needed by noting the Review ID.

3. Click on the Orange icon with a hammer to begin the adjudication for a given patient.

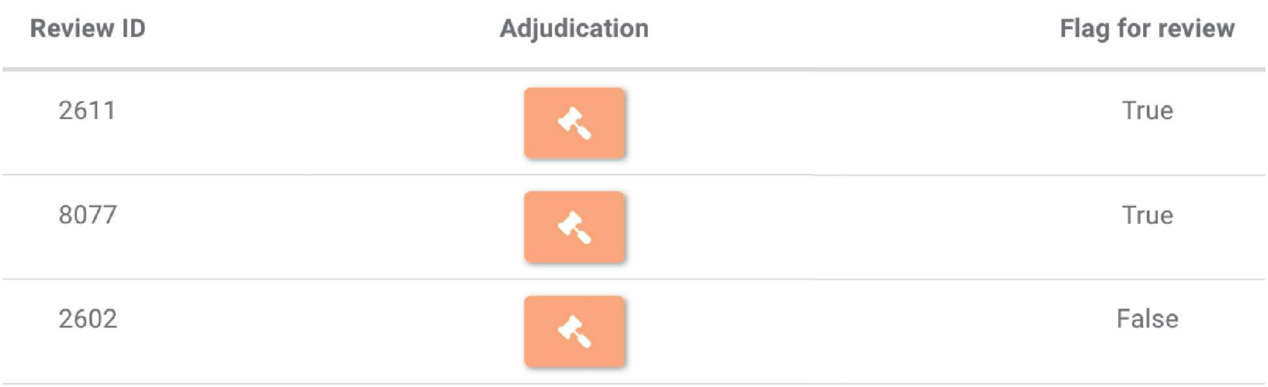

4. In the first section, you will see the patient's demographic information such as: age, sex, reason for admission, frailty, GFR, living arrangements prior to admission, discharge location, readmissions to ER/hospital (if applicable), death details (if applicable) and interviewer notes (if applicable). There is a scrambled patient ID so you cannot tell the site nor the intervention status. **The following screenshots are simulated patient data and do not constitute a real patient.**

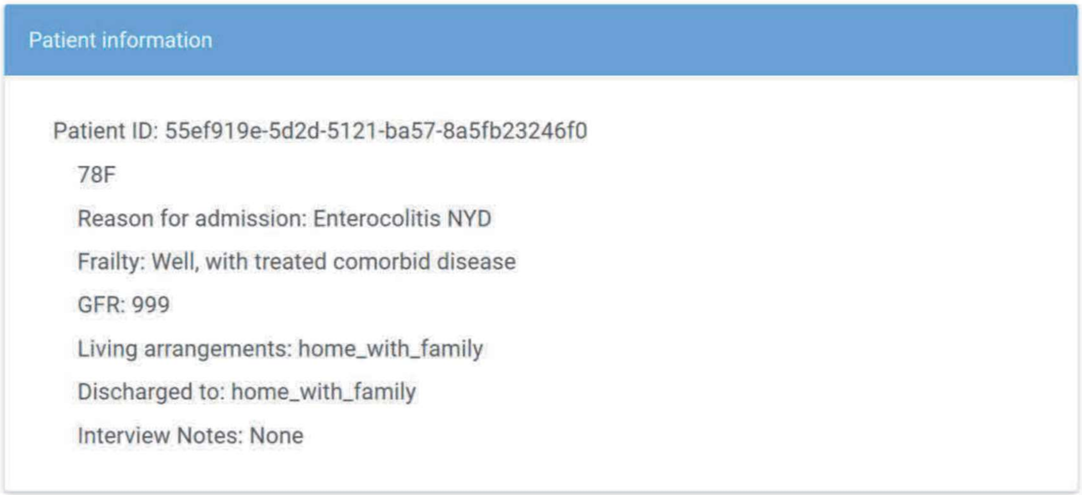

Note: if the patient was readmitted to the hospital within 30 days, the research assistant will have completed something called a "**short-form interview**" and you will see this message:

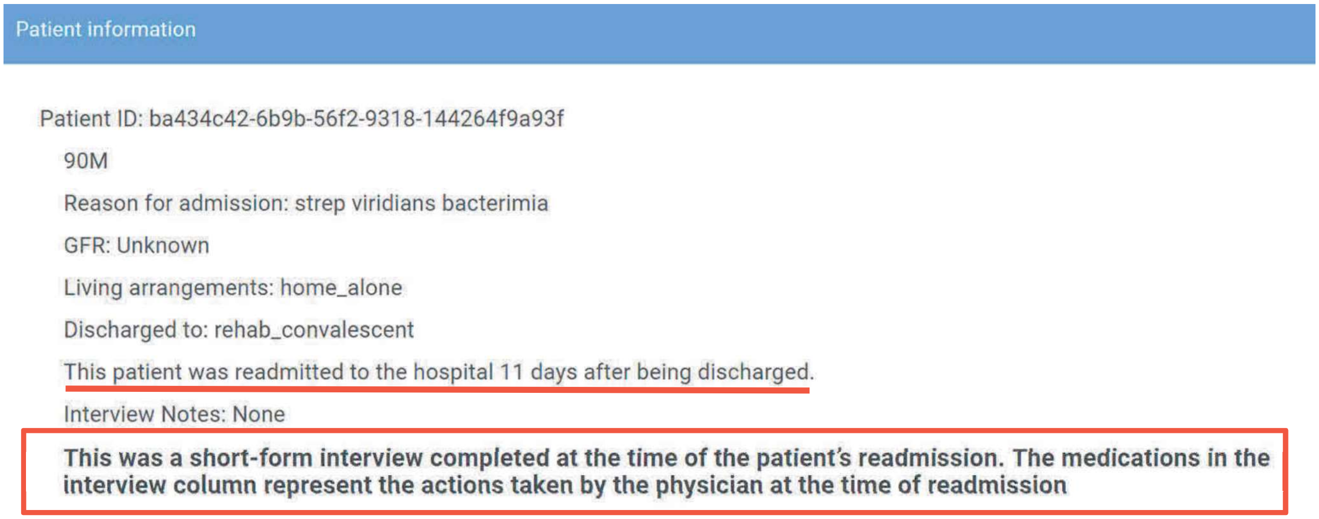

5. Under PMH (past medical history), you will see all conditions selected by the Research Assistant at the time of admission.

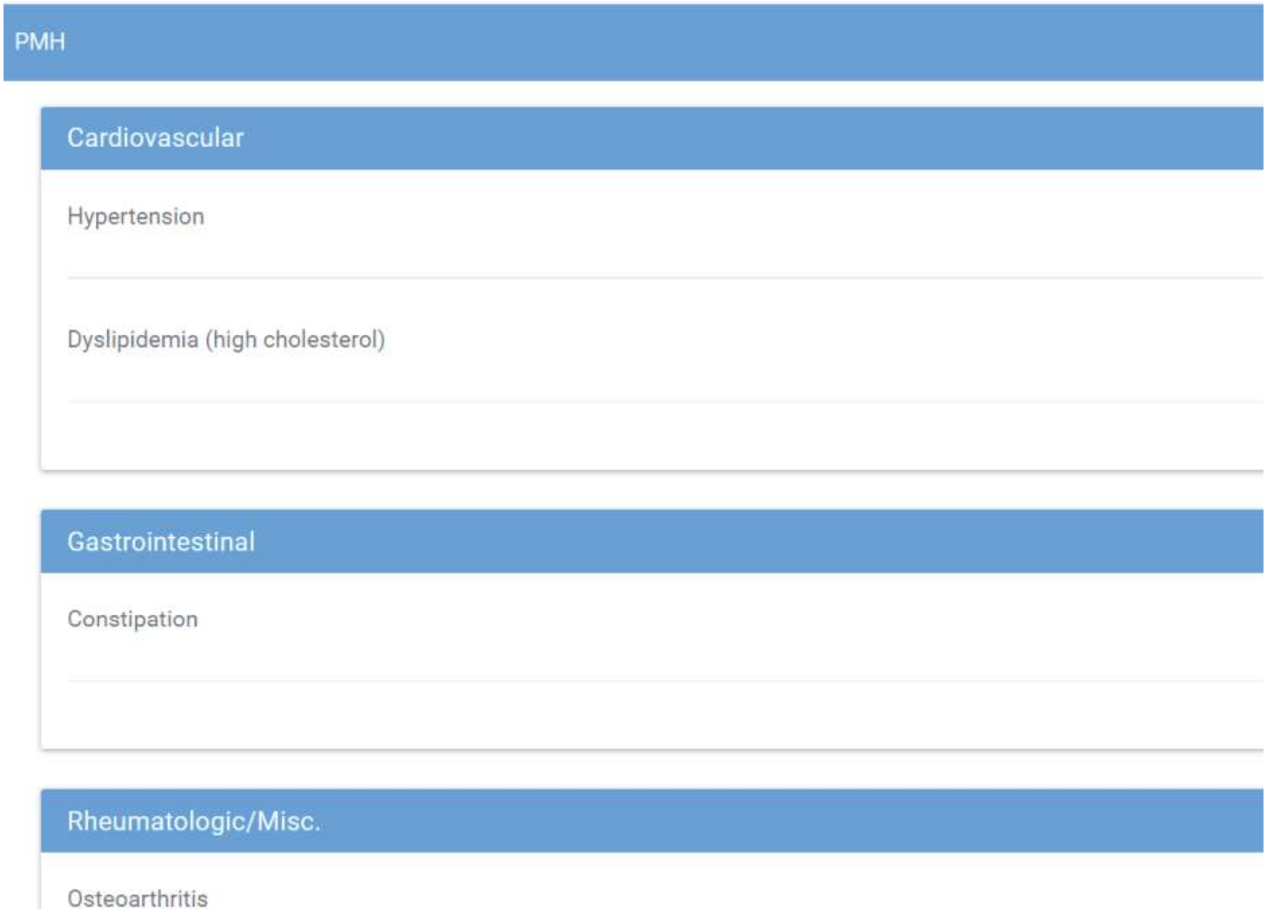

6.

e

dical History section, you will be able to view the following information recorded by the Interviewer during the 30-day post-discharge phone call:

a) Conditions (new or worsening)
b) Contact with healthcare system
c) Medications at the time of the interview

a. Conditions
This section shows new or worsening conditions reported by the patient since discharge.

Conditions

Condition	New/Worse Condition (Interview)	Details	Days Post Discharge	How long did it last?	Adverse Event	ADE
pneumonia	Worse	problem breathing, went to the ER where he was initially told he had an infection. was in the ER 2 days	29		Adverse Event ☐	Adverse Drug Event ☐

b. Contact with healthcare system

In this section, visits to the ER, clinic appointments and readmissions to the hospital are recorded.

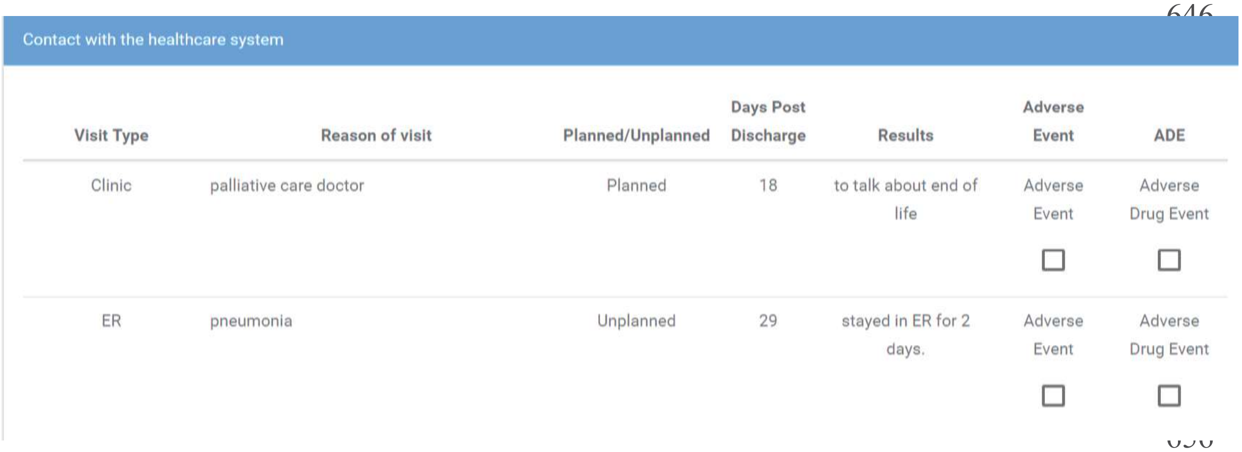

Contact with the healthcare system

Visit Type	Reason of visit	Planned/Unplanned	Days Post Discharge	Results	Adverse Event	ADE
Clinic	palliative care doctor	Planned	18	to talk about end of life	Adverse Event ☐	Adverse Drug Event ☐
ER	pneumonia	Unplanned	29	stayed in ER for 2 days.	Adverse Event ☐	Adverse Drug Event ☐

*Note that if an event occurred more than 30 days post-discharge this will be indicated.

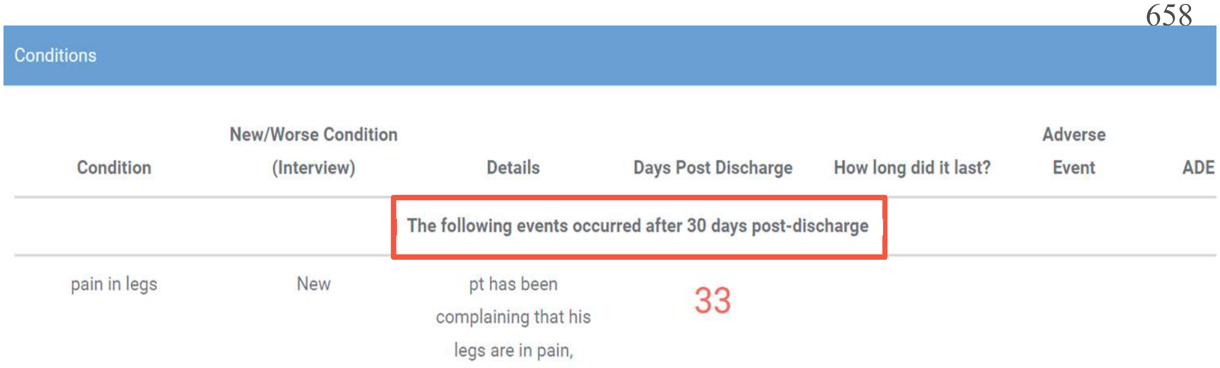

Conditions

Condition	New/Worse Condition (Interview)	Details	Days Post Discharge	How long did it last?	Adverse Event	ADE
		The following events occurred after 30 days post-discharge				
pain in legs	New	pt has been complaining that his legs are in pain,	33			

c. **Medication history**

- The **Admission** column on the left shows the medication and dosage the patient was taking prior to admission.
- The **Discharge** column shows what action (continued, modified, stopped) was taken with these medications at the time of discharge from the hospital (from med reconciliation).
- The **Interview** column contains changes in meds between discharge and the time of the interview, including medications stopped, restarted, or newly started post-discharge.

- If a medication was continued, it will remain **green** in each column *(see clopidogrel)*.
- If a medication was modified at discharge from the hospital, the medication will be **orange** in the **Discharge** column. *Note that for a medication change at discharge, you will need to read the new dosage to see if it is an increase or decrease*. *The field that was modified will be indicated in bold*. If the medication in the interview column is green, this means it was continued at the modified discharge dose *(see nitroglycerin)*.
- If the medication was modified following discharge, the medication will be **orange** in the **Interview** column. The arrow will indicate if this is an increase or decrease from discharge (*see ciclesonide*).
- If a medication is stopped at discharge, it will no longer appear in that column *(see pantoprazole)*.
- If a medication was stopped post-discharge, this will be indicated in **red** *(see tamsulosin)*.
- For changes recorded by the interviewer, by who, when and why the change was made will be indicated if applicable.
- Medications started at discharge from the hospital that were not taken prior to admission will not appear in the Admission column *(see acetaminophen)*.
- medications that were started after the discharge from hospital will appear in **yellow**. If available, the information regarding why and when it was prescribed will be indicated *(see finasteride)*. If no reason for starting could be elucidated from the interview it will say "none"
- Reminder: If a short-form interview was completed by the RA for a 30-day readmission, the medications in the Interview column represent the actions taken by the team at the time of readmission.

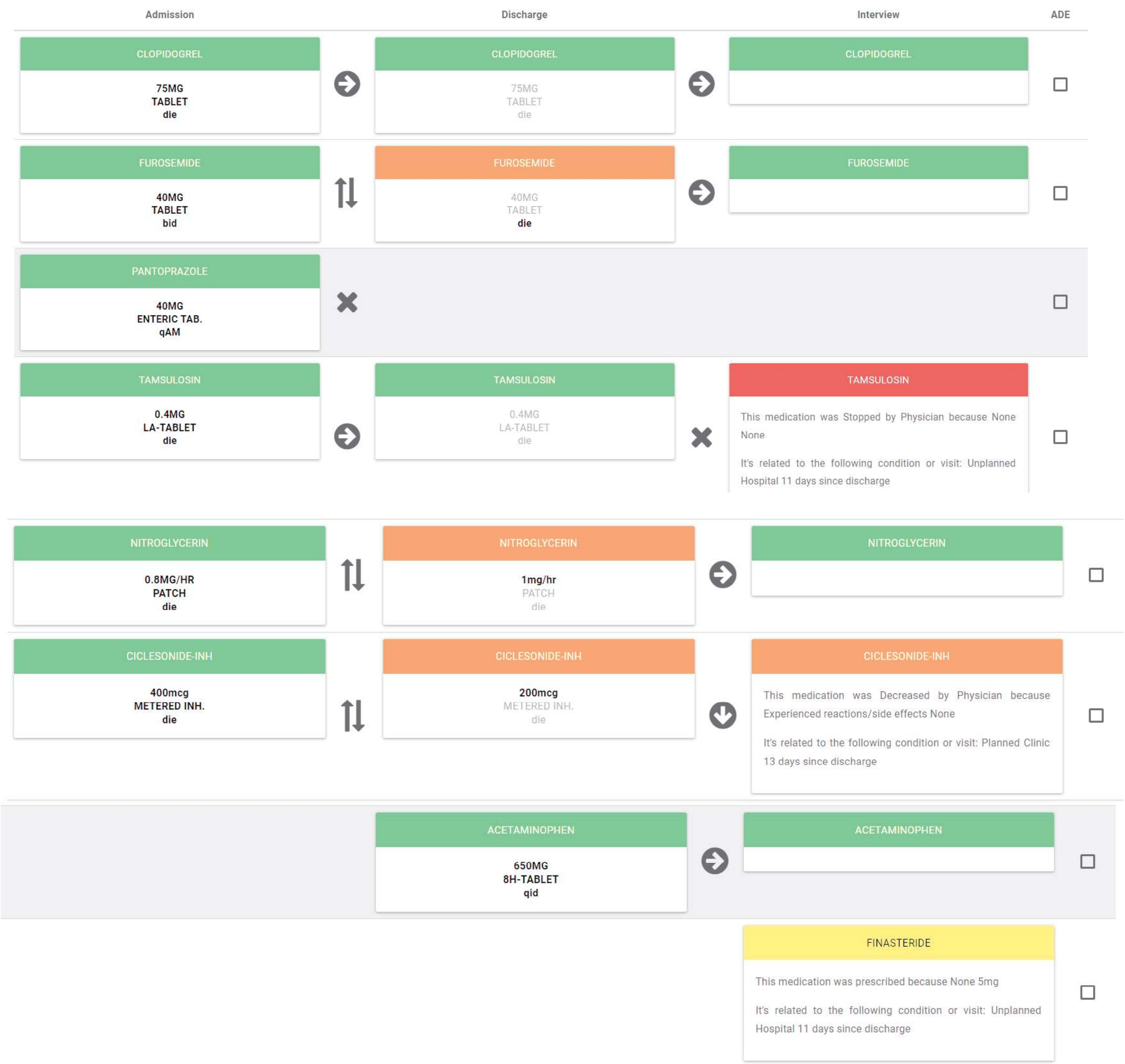
Admission
Discharge
Interview
ADE
CLOPIDOGREL
75MG
TABLET
die
CLOPIDOGREL
75MG
TABLET
die
CLOPIDOGREL
FUROSEMIDE
40MG
TABLET
bid
FUROSEMIDE
40MG
TABLET
die
FUROSEMIDE
PANTOPRAZOLE
40MG
ENTERIC TAB.
qAM
TAMSULOSIN
0.4MG
LA-TABLET
die
TAMSULOSIN
0.4MG
LA-TABLET
die
TAMSULOSIN
This medication was Stopped by Physician because None None
It's related to the following condition or visit: Unplanned Hospital 11 days since discharge
NITROGLYCERIN
0.8MG/HR
PATCH
die
NITROGLYCERIN
1mg/hr
PATCH
die
NITROGLYCERIN
CICLESONIDE-INH
400mcg
METERED INH.
die
CICLESONIDE-INH
200mcg
METERED INH.
die
CICLESONIDE-INH
This medication was Decreased by Physician because Experienced reactions/side effects None
It's related to the following condition or visit: Planned Clinic 13 days since discharge
ACETAMINOPHEN
650MG
8H-TABLET
qid
ACETAMINOPHEN
FINASTERIDE
This medication was prescribed because None 5mg
It's related to the following condition or visit: Unplanned Hospital 11 days since discharge

7. In the Analysis section, you will select whether you believe the patient had an

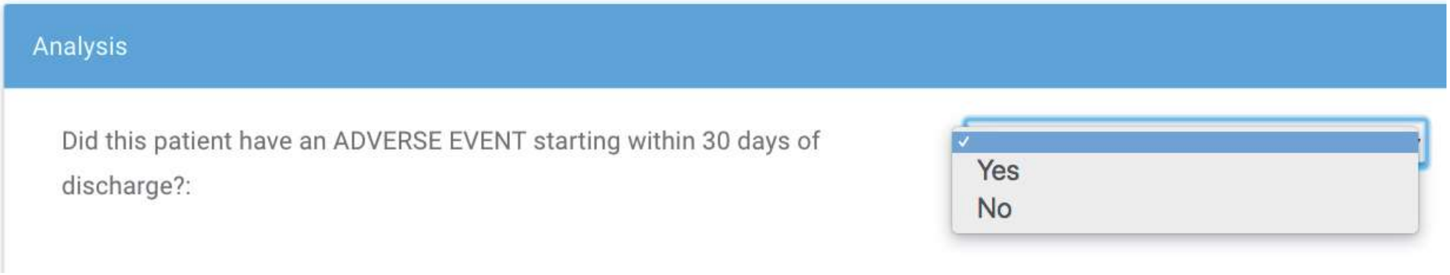

8. ANY **Adverse Event** (may or may not be drug-related) within 30 days of discharge.

If NO, then click "Complete this Adjudication". You will be asked to confirm that you

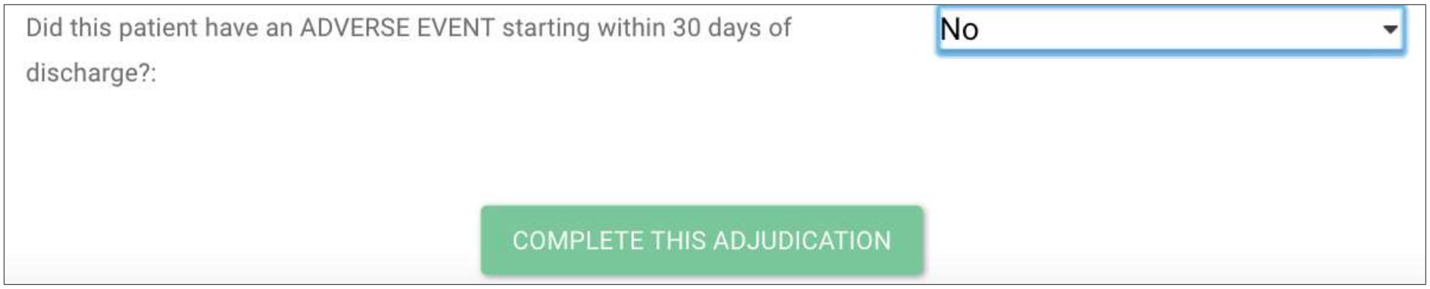

have completed the adjudication. If not, then select Cancel.

If YES, **scroll up** and select the Adverse Event checkbox on the far-right side next to the appropriate condition(s) or visit(s) you believe is related to the event.

*Note: <u>Do not select any events beyond 30 days</u> unless you believe they clearly started within the 30-day period.

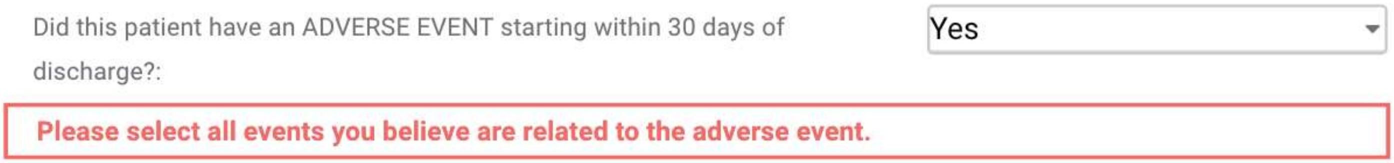

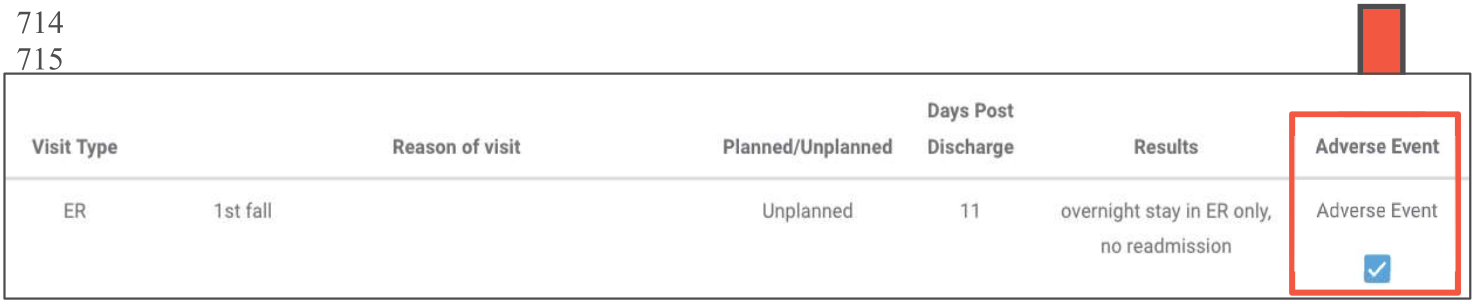

Visit Type	Reason of visit	Planned/Unplanned	Days Post Discharge	Results	Adverse Event
ER	1st fall	Unplanned	11	overnight stay in ER only, no readmission	Adverse Event

9. Next, select whether you think the patient's event was an **adverse DRUG event**. Select an option from 1 to 6. An ADE is adjudicated as positive if 5 or 6 is selected.

   If option 1 to 4 is selected (not due to an adverse drug event), complete the adjudication. If option 5 or 6 is selected, **scroll up** and check off the ADE checkbox next to any and all condition(s), visit(s), and medication(s) you think are responsible.

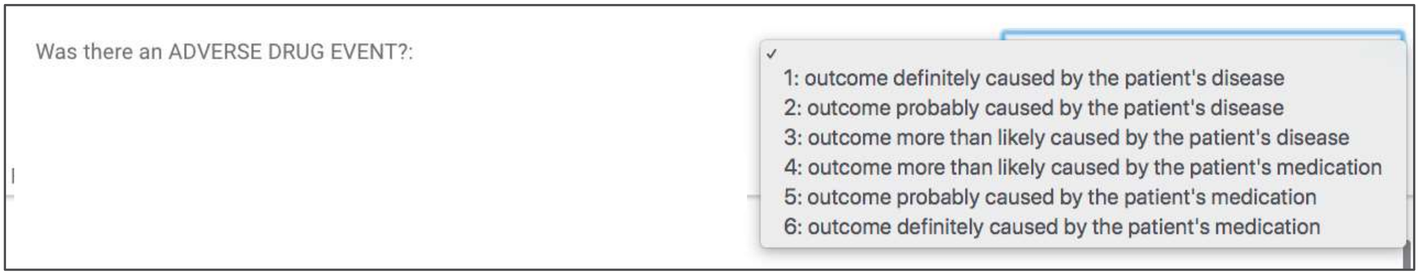

10. Select whether or not you think at least one of the events was preventable (ex. a patient is newly started on quetiapine at discharge and returns to the ED a few days later with delirium; this could have been prevented if quetiapine had not been started.)

11. Select whether at least one event was ameliorable. (Ex. a patient has gastrointestinal hemorrhage and they are taking aspirin and coumadin. There is no obvious indication for taking both on an ongoing basis from examining the past medical history. Both drugs were continued at discharge. Aspirin could have been stopped.)

12. Select whether the ADE was related to a withdrawal event (e.g. secondary to reducing, tapering, or stopping a medication)?

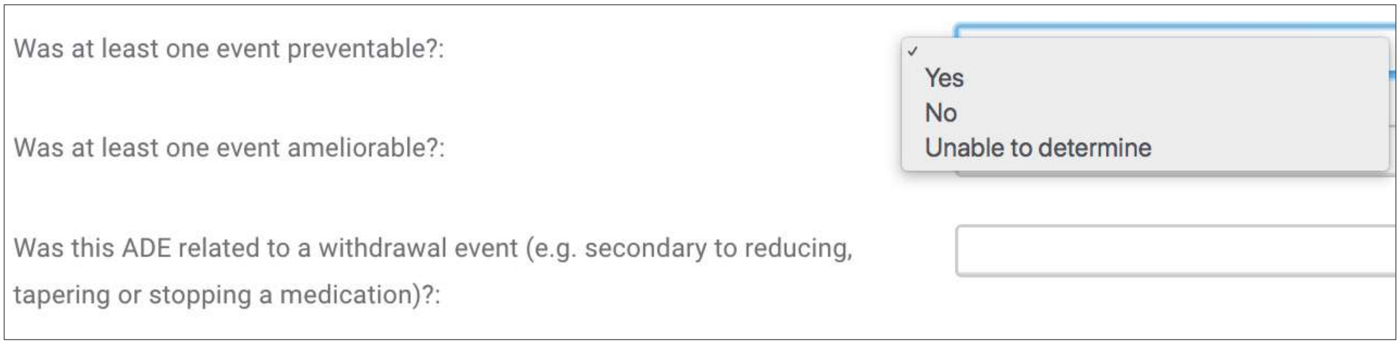

13. In some cases, the ADE is completely unrelated to the hospitalization. You should next select if the ADE is related to the hospitalization or not. Most ADEs are tied back to the hospitalization in some manner of speaking, but occasionally they are totally unrelated. Here is a real-life example: A patient visits their family doctor 10 days after hospital discharge and is newly prescribed gabapentin for leg cramps. At the time of the interview they state they have had new swelling in their legs since starting gabapentin and went to the ER for that. This is an adverse drug event. It is an ADE that is completely unrelated to the hospitalization, however. Here is another example: The

patient develops a rash on their leg and is given cefadroxil by their cardiologist at 20 days post discharge. They then develop anaphylaxis and must go to the ER. This is an adverse drug event. But it is completely unrelated to the hospitalization.

Once you have answered all the questions and selected all the appropriate checkboxes, click on the “Complete this Adjudication” button at the bottom of the page.

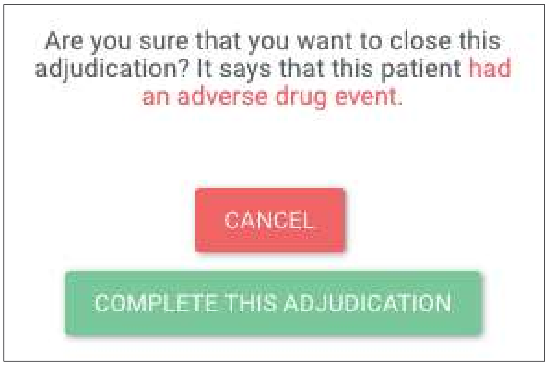

Examples of AEs

1. A patient is readmitted to the hospital at 26 days post hospital discharge with shortness of breath and cough. This patient had an adverse event. On the Likert scale for ADE, this was judged to be more than likely related to the patient’s medical conditions as opposed to one of their medications. Therefore, it is adjudicated as an adverse event, but not an ADE.
2. A patient takes aspirin chronically. The prescription remains unchanged at discharge from hospital. At 29 days post hospital discharge the patient presents to the ER with blood per rectum and they are told it is not serious and sent home. This is an adverse event (presents to the ER within 30 days post discharge). This was adjudicated as a 4/6 on the Likert scale (more than likely related to the medication). This is not certain enough to qualify as an ADE for this study. Therefore, the file is closed as an adverse event, but not as an ADE. We will do a planned secondary analysis that includes 4/6 adjudications. For the primary outcome of this study only 5/6 or 6/6 is classified as an ADE.
3. A patient with COPD is discharged and represents to the ER five days after discharge with dyspnea and in need of supplemental oxygen This is an adverse event but not an adverse drug event (caused by underlying condition)
4. A patient develops shortness of breath, and pulmonary edema that requires a chest x-ray, Lasix, and nitro emergency treatment at their residence. Upon review of the medications, there were no changes at discharge that may have contributed. This adverse event was probably caused by the patient’s underlying condition.

Examples of ADEs

1. A patient is prescribed **sertraline** newly at hospital discharge and begins to develop substantial **nausea and anorexia** in the 30-days post hospital discharge after starting sertraline. This is an adverse event and is ALSO AN ADE. It is probably related to the sertraline and was adjudicated as a 5/6 on the Likert scale. These are known side effects of the medication and started in a consistent timeframe.
2. A patient is taking chronic **pantoprazole**. At hospital discharge you see they are newly started on magnesium pills. One week after discharge the patient has a **traumatic fall** and in the ER, they tell her it is because she has **critically low magnesium levels**. She receives IV magnesium 3 times over the next month. All the while she has been on pantoprazole. This is an ADE. Hypomagnesemia is a recognized side effect of PPI therapy and can occur at any time (a paradoxical reaction) that the patient is taking the medication. This was adjudicated as a 5/6 on the Likert scale. There was no history of cancer or chemotherapy or other past medical condition to explain the low magnesium.
3. A patient is diagnosed with giant cell arteritis and started on **prednisone 50 mg po daily** and returns with upper GI bleeding the week following their discharge with **an ulcer** that requires cauterizing on gastroscopy. This is ADE secondary to prednisone. This is a known side effect of the medication.
4. A patient is **newly started on trazodone** for sleep at hospital discharge and returns to the hospital 1-2 weeks later with a **fall and bleeding in the head**. Sedation and falls are known side effects of medications with sedating properties prescribed for sleep. This was scored as 5/6 on the Likert scale.
5. At discharge from the hospital a patient is **started on asa** in addition to their usual clopidogrel. They are also **started on codeine 60 mg po QID**. Following this the patient develops **nausea and recurrent vomiting** for several days. The patient's son researches on the internet and stops the codeine and the patient feels better/vomiting diminishes in frequency and severity. This is an adverse drug event.

Examples that DO NOT qualify as an Adverse Event:

1. A patient is discharged, and they have a **TIA at 31 days post hospital discharge**. REASON: For the primary study outcome we are only looking at events that occur up to 30-days post discharge. We do not include events that start after 30 days. For this to qualify as an adverse event it would need to have started on day 30 or earlier.
2. A patient is admitted with the principal diagnosis of stroke. After discharge they have several **planned visits** with their doctors including their ophthalmologist who performs an intraocular injection for fluid build-up in the eye. This is not an adverse event.
3. A patient is discharged from the hospital following admission for pneumonia. 26 days after discharge they develop **a sore throat** and see their family doctor. This is not an adverse event.
4. A patient is discharged following an admission for bacteremia. They develop **diarrhea** 29 days after discharge which resolves one day later on its own. This was not considered an adverse event or an adverse drug event (once medications and conditions reviewed).

5. A patient is readmitted to the hospital **32 days** after hospital discharge after being found on the floor at home with a temperature of 29C and altered level of consciousness. This cannot be adjudicated as an adverse event as it took place more than 30 days after hospital discharge unless you believe it started before day 30. Please do not adjudicate anything that takes place beyond 30 days post discharge unless it clearly starts within 30 days of discharge.

Examples that are NOT classified as an ADEs:

1. A patient takes aspirin for ischemic heart disease and is diagnosed with mouth/throat cancer. They start bleeding from their mouth cancer following hospital discharge and need to go the ED and receive a blood transfusion. This is an adverse event. It was scored as 4/6 on Likert scale (the outcome was more than likely caused by the patient's drug). In this study, this will NOT be classified as an ADE for the primary outcome. In this case, it is not clear how much the disease contributed to the bleeding as opposed to the medication. Certainly, the aspirin contributed to the bleeding. We will do a preplanned sensitivity analysis including 4/6 along with 5 and 6/6 scores.
2. A patient sees his cardiologist and family doctor for planned visits at 15 days post-discharge. The cardiologist decreases the furosemide from 40 mg po daily to 20 mg po daily and arranges to see the patient again in follow-up. This is normal clinical titration of furosemide and is not an adverse drug event.
3. A patient has their diabetes medications decreased at hospital discharge. When they see their physician in follow-up the medications are further adjusted and some of the stopped medications are restarted. This is not an adverse drug event.

Section 8 – Assessment of Safety

8.1 Specification *of safety parameters*

During adverse drug event adjudication, adverse drug events due to withdrawal of a drug (stopping, tapering, holding, accidental failure to represcribe or deprescribing) are captured in the case report form. Any adverse drug event that is from deprescribing will be reported as a secondary outcome (adverse drug withdrawal events).

8.2 The methods and timing for assessing, recording, and analysing safety parameters.

Throughout the study, a study email is routinely monitored for reporting of any adverse events. Each deprescribing report contains the study email and the deprescribing report prompts the recipient to contact the study team in the event of any perceived adverse event. We will also have monthly meetings with study teams to obtain feedback on the tool and solicit any perceived adverse events. Finally, we will capture adverse drug withdrawal events and evaluate them over the course of the study and associated with the intervention.

8.3 Procedures for eliciting reports of and for recording and reporting adverse event and intercurrent illnesses.

All deprescribing reports contain the contact information for the principal investigator/study email and the treating team is encouraged to contact the study team in the event of an adverse event. Otherwise, adverse events are collected and analyzed at the end of the study and are captured in the case review file as adverse drug withdrawal events.

8.4 The type and duration of the follow-up of subjects after adverse events.

Subjects who have an adverse event receive usual hospital care. Follow-up is limited to the 30-day telephone screening questionnaire. No long-term follow-up after an adverse event is planned other than usual care (presenting as needed to a usual treating physician, urgent care service, or emergency department).

Section 9 – Statistics/Statistical Analysis Plan

9.1a *A description of the statistical methods to be employed, including timing of any planned interim analysis(ses).*

Baseline characteristics will be expressed as numbers and percentages for categorical variables and median and interquartile range (IQR) for continuous variables. Differences between control and intervention baseline characteristics will be compared by chi-square or rank-sum as appropriate (for descriptive purposes since this is a cluster randomized trial and there may be chance imbalances due to case-mix during the intervention and control periods).

All analysis will be by intention to treat except where otherwise noted.

Primary Outcome - Adverse Drug Events

A mixed-effects logistic regression model will be used for the outcome of adverse drug events (>=5 on Leape-Bates scale) which controls for intervention, time period, and number of baseline potentially inappropriate medications as fixed effects and cluster as a random effect. This model will be used to estimate adjusted risk differences[34].

A sensitivity analysis will be conducted (supplemental) using a Leape-Bates Scale of >=4 for the definition of adverse drug events.

This analysis will be conducted on patients who have consented to, and participated in, the post-discharge follow-up.

Secondary Outcome(s)

- *All adverse events within 30-days post discharge*

A mixed-effects logistic regression model will be used for the outcome of adverse events (whether or not ADE-related) which controls for intervention, time period, and number of baseline potentially inappropriate medications as fixed effects and cluster as a random effect. This model will be used to estimate adjusted risk differences[34]. This analysis will be conducted on patients who have consented to, and participated in, the post-discharge follow-up.

- *Adverse drug withdrawal events within 30-days post discharge*

As these are expected to be a rare subset of adverse events, we will provide a comparison of the number of these events in the control and the intervention and the proportion they represent of total adverse drug events. There will be no statistical comparison. This analysis will be conducted on patients who have consented to, and participated in, the post-discharge follow-up.

- *Proportion of patients with one or more PIMs stopped at discharge*

A mixed-effects logistic regression model will be used for the outcome of stopping 1 or more PIM which controls for intervention, time period, and number of baseline potentially inappropriate medications as fixed effects and cluster as a random effect. This model will be

used to estimate adjusted risk differences[34]. This analysis will be conducted on patients who are taking 1 or more PIM at admission and who are discharged alive from a study unit.

- *ER Visit/Readmission within 30-days of hospital discharge*

A mixed-effects logistic regression model will be used for the outcome of ER visit or readmission post discharge which controls for intervention, time period, frailty status, long-term care residence, age, gender and number of baseline potentially inappropriate medications as fixed effects and cluster as a random effect. This analysis will be conducted on patients who have consented to, and participated in, the post-discharge follow-up.

- *Deaths within 30-days of hospital discharge*

A mixed-effects logistic regression model will be used for the outcome of death post discharge which controls for intervention, time period, frailty status, HOMR score and number of baseline potentially inappropriate medications as fixed effects and cluster as a random effect. This analysis will be conducted on patients who have consented to, and participated in, the post-discharge follow-up.

- *Falls within 30-days of hospital discharge*

A mixed-effects logistic regression model will be used for the outcome of fall post discharge which controls for intervention, time period, frailty status, long-term care residence, age, gender and number of baseline potentially inappropriate medications as fixed effects and cluster as a random effect. This analysis will be conducted on patients who have consented to, and participated in, the post-discharge follow-up.

- *Absolute number of medications at discharge*

A generalized linear regression model will compare the number of medications at discharge controlling for intervention, time period, and the number of baseline medications as fixed effects and cluster as a random effect. This analysis will be conducted on all patients who are discharged alive on a study unit (see above re transitions of care).

- *Absolute number of medications at 30 days post-discharge*

A generalized linear regression model will compare the number of medications at 30-days post discharge controlling for intervention, time period, and the number of baseline medications as fixed effects and cluster as a random effect. This analysis will be conducted on patients who have consented to, and participated in, the post-discharge follow-up.

- *Absolute number of PIMs at discharge*

A generalized linear regression model will compare the number of PIMs at discharge controlling for intervention, time period, and the number of baseline PIMs as fixed effects and cluster as a

random effect. This analysis will be conducted on all patients who are discharged alive on a study unit (see above re transitions of care).

- *Proportion of medications that remain deprescribed at 30 days post-discharge*

For drugs which were deprescribed at discharge, the proportion which remained prescribed at 30-days (without resuming) will be given for the control and intervention periods without statistical testing. This analysis will be conducted on patients who have consented to, and participated in, the post-discharge follow-up.

- *30-day post-discharge sleep questionnaire responses (Patient-Reported Outcomes Measurement Information System Sleep Disturbance Form 4a)*

For individual elements of the score, the number and proportion of each response will be presented in tabular form for pre-hospital and post-hospital results, stratified by intervention for all respondents. The total score will be transformed to a T-score using the standardized table for adults and the median T-scores and interquartile ranges will be presented in tabular form for those respondents who completed both questionnaires and will be stratified by intervention. The Wilcoxon matched pairs sign rank will be used to compare prehospital scores to post discharge scores within intervention and control respectively.

- *30-day self-reported health-related quality of life (EQ-5D-5L and Visual Analog Scale)*

Among those who complete the post discharge interview and who are able to provide responses to the EQ-5D-5L questionnaire, the raw scores will be converted into estimates of quality of life using a validated Canadian time-trade off value set[35]. They will be tabulated with median values and interquartile ranges along with the median and IQR for the visual analog scale. A simple comparison between the intervention and control for these measures will use Wilcoxon rank sum.

Sensitivity analyses as indicated above.

9.1b *A description of the post-hoc statistical methods to address imbalances between the cluster and the intervention groups*

We performed 5 post-hoc sensitivity analyses to address the low number of clusters in the study: 1) an analysis adjusting for patient factors that differed between groups by P<0.01; 2) treating clusters as a fixed-effect[36]; an analysis with 3) a random and 4) fixed effect for hospital (n=11, in lieu of cluster) and 5) using permutation, a non-parametric method of evaluating for the weighted within period effect size[37, 38].

9.2 *The number of subjects planned to be enrolled (sample size calculation)*

The expected proportion of medical patients who will experience an ADE after discharge is between 10 and 19%[19, 29, 39, 40]. For our power calculation we have used the midpoint, 15%. Type

1 error will be 5% and type 2 will be 20%. The cluster correlation expected is no more than 0.03[8]. We wish to show an absolute decrease of four percent. An 8400-patient trial comprising 6 clusters who each recruit 200 patients per cluster-period will have 80% power to detect a change in ADE of from 15% to 11.2% or lower (an absolute risk reduction of 3.8%, number needed to treat 27). Implementation of this power calculation used the Stata function "steppedwedge[41]" and followed the Hussey and Hughes approach[42].

Revised prior to the recruitment of the first 200 patients to any cluster to:

Three clusters will be created based on geographical location in Canada (Quebec, Ontario, Western Canada). These clusters are expected to enroll 500 subjects per time period for a total of 1 baseline and 3 randomized time-periods. The total number of patients will be 6000. With an estimated 15% adverse drug event rate, this will yield at least 80% power to detect an absolute difference of 4.8% (NNT 21) with an alpha of 0.05 and varying intra-cluster correlation ranging from 0.01 to 0.05.

9.3 *The level of significance to be used.*

An alpha of 0.05 will be used. There will be no adjustment for multiple comparisons.

9.4 *Criteria for the termination of the trial.*

There are no stopping criteria for this trial as the timing of ADE adjudication and the order of randomization of the cluster trial design does not permit an implementable interim analysis.

9.5 *Procedure for accounting for missing, unused, and spurious data.*

Significant attempts will be made to have complete data. In the event of missing data, complete case analysis or multiple imputation will be performed as are appropriate to the situation.

9.6 *Procedures for reporting any deviation(s) from the original statistical plan (any deviation(s) from the original statistical plan should be described and justified in protocol and/or in the final report, as appropriate).*

Deviations will be clearly highlighted and the final clinicaltrials.gov submission will be updated accordingly.

9.7 *The selection of subjects to be included in the analyses (e.g., all randomized subjects, all dosed subjects, all eligible subjects, evaluable subjects).*

All subjects who consent and receive a deprescribing report while in hospital and who survive to hospital discharge will be analyzed for the primary outcome (which occurs within 30-days of hospital discharge). Subjects who die in hospital will not be analyzed for the primary outcome as they cannot experience the primary outcome.

For measures of efficacy on deprescribing at discharge, the population of interest will be all subjects who have 1 or more potentially inappropriate medication identified at baseline and who

are discharged alive from a study unit regardless of whether or not they complete the post-discharge interview.

Section 10 – *Direct Access to Source Data/Documents*

Medical records will be abstracted by trained personnel directly from clinical documents and input into the MedSafer tool which also functions as the study database. No copies of source data will be maintained outside of the patient's medicolegal chart. Redacted patient charts will be transmitted securely to the coordinating centre monthly as described in Section 11; however, these will be securely destroyed once quality assurance checks have occurred.

Section 11 – Quality Control and Quality Assurance

Each clinical site will perform internal quality management of study conduct, data collection, documentation, and completion.

Quality control (QC) procedures will be implemented beginning with the data entry system and data QC checks that will be run on the database will be generated. Any missing data or data anomalies will be communicated to the site(s) for clarification/resolution.

Research assistants will be trained during a two-week period by an assistant who worked on the pilot MedSafer study. During the training period anonymous case files will be used for teaching of data collection and data entry. A training manual will be generated for research assistants with frequently asked questions. Each month each site research assistant will submit 5 anonymous case files for QC check of data entry.

Section 12 – Ethics

This study will be conducted in accordance with the principles of Good Clinical Practice and the Declaration of Helsinki. All study sites will obtain approval from their ethics review boards and follow local policies and standard operating procedures.

All personnel involved in the design and conduct of the research involving human participants will receive the required education on the protection of human research participants prior to the start of this project. The study consent form will discuss the study, including the risks and benefits of participation. Informed consent will be obtained from all participants before any study procedures are initiated. The consent form contains a description of the purpose and procedures, risks, procedures to minimize them, and possible benefits. Participants will be assured that participation in the study is completely voluntary, and that they are free to withdraw consent at any time and discontinue participation without prejudice to their current or future medical care. Except for blinding during the control phase, the objectives of the project, all the requirements for participation, and any possible discomforts and risks will be clearly explained to the participants verbally and in writing.

All participants (or their proxy) must sign a consent document indicating their consent, approved by the site research ethics board, before they can participate in the study.

Participants who wish to ask additional questions about the study procedures or risks and benefits of the study will be offered this opportunity by email, phone call or meeting with the study team (depending on their preference).

We will seek a waiver of consent for the intervention from provinces that will allow this, which is considered best practice (medication reconciliation with identified opportunities for deprescribing) but will seek consent for the 30-day follow-up phone interview from all participants.

Section 13 – Data Handling and Record Keeping

All study findings and documents will be confidential. The investigators and other study personnel must not disclose such information without prior written approval from the Principal Investigator. Participant confidentiality will be strictly maintained to the extent possible under the law and as required by Good Clinical Practice. Identifiable information will be removed from any published data.

The web server housing the study tool is behind a reverse web proxy, which allows for the server to remain anonymous to users and serves as an additional line of defense in case of an attack. In other words, the server itself is completely hidden from everyone with the only entry point being the login page of the web application.

The login page of the application is written in Python using the Flask framework. These are both mature and well-tested languages and frameworks, which provide plenty of security mechanisms to prevent any unauthorized access. All the communication is encrypted using the most advanced algorithms (HMAC and SHA1 precisely) and every single page requires the user to be logged in.

Accounts will only be given to the specifically hired research assistants at each site, each of whom will sign a confidentiality agreement with their institution. Account management will be performed by the principal investigators based on the requests for access from the individual sites. Users will be required to choose a password of suitable complexity and security based on best password practices.
https://security.web.cern.ch/security/recommendations/en/passwords.shtml

The Oracle database is an enterprise solution that provides strong security. Permissions to the application's data will be granted only to the database administrator and to researchers for the hospital that they should have access to only.

Only RI-MUHC personnel will have access to MUHC patient's data within the application with the exception of the data safety monitoring committee and the study's principal investigators who will have access to data for the whole study (study PIs and project manager will have access to all data in case of emergency or need to audit). The instance is also isolated behind a VPN, which prevents outside users from trying to access it.

Section 14 – Financing and Insurance

This study is funded by the Canadian Institutes for Health Research and is investigator-initiated and derives no income from industry sources.

Each participating center will make sure that their research institute has the correct insurance policies for conducting investigator-initiated randomized controlled trials and that each

participating physician-investigator has appropriate malpractice insurance for the Province in which they practice.

Section 15 – Publication Policy

We will publish the trial in a peer-reviewed journal and will make the manuscript open access (whether positive or negative). The choice of a peer-reviewed journal will be dependent on the study findings but will likely be related to the field of general medicine. All investigators who meet the ICJME criteria for authorship will have the opportunity to be co-authors on the manuscripts.

Section 16 – Making data available for secondary use

After publication, we will make available a curated anonymous dataset with the data points needed to carry out a reproduction of the primary findings of the trial manuscript. We will also plan to make the remainder of the trial data available for secondary use by investigators subject to data sharing agreements executed under Quebec law. Such data can be obtained via a request to the principal investigator Dr. Todd Lee at todd.lee@mcgill.ca.

Section 17- References

1. Kuijpers MA, van Marum RJ, Egberts AC, Jansen PA. Relationship between polypharmacy and underprescribing. *Br J Clin Pharmacol*. Jan 2008;65(1):130-3. doi:10.1111/j.1365-2125.2007.02961.x
2. Linton A, Garber M, Fagan NK, Peterson MR. Examination of multiple medication use among TRICARE beneficiaries aged 65 years and older. *J Manag Care Pharm*. Mar 2007;13(2):155-62. doi:10.18553/jmcp.2007.13.2.155
3. Boyd CM, Darer J, Boult C, Fried LP, Boult L, Wu AW. Clinical practice guidelines and quality of care for older patients with multiple comorbid diseases: implications for pay for performance. *Jama*. Aug 10 2005;294(6):716-24. doi:10.1001/jama.294.6.716
4. Astrand B, Astrand E, Antonov K, Petersson G. Detection of potential drug interactions - a model for a national pharmacy register. *Eur J Clin Pharmacol*. Sep 2006;62(9):749-56. doi:10.1007/s00228-006-0143-x
5. Hamilton H, Gallagher P, Ryan C, Byrne S, O'Mahony D. Potentially inappropriate medications defined by STOPP criteria and the risk of adverse drug events in older hospitalized patients. *Arch Intern Med*. Jun 13 2011;171(11):1013-9. doi:10.1001/archinternmed.2011.215
6. Steinman MA, Hanlon JT. Managing medications in clinically complex elders: "There's got to be a happy medium". *Jama*. Oct 13 2010;304(14):1592-601. doi:10.1001/jama.2010.1482
7. Poudel A, Peel NM, Nissen L, Mitchell C, Gray LC, Hubbard RE. Potentially inappropriate prescribing in older patients discharged from acute care hospitals to residential aged care facilities. *Ann Pharmacother*. Nov 2014;48(11):1425-33. doi:10.1177/1060028014548568
8. Tamblyn R, Huang AR, Meguerditchian AN, et al. Using novel Canadian resources to improve medication reconciliation at discharge: study protocol for a randomized controlled trial. *Trials*. Aug 27 2012;13:150. doi:10.1186/1745-6215-13-150
9. Marengoni A, Pasina L, Concoreggi C, et al. Understanding adverse drug reactions in older adults through drug-drug interactions. *Eur J Intern Med*. Nov 2014;25(9):843-6. doi:10.1016/j.ejim.2014.10.001
10. Hamilton HJ, Gallagher PF, O'Mahony D. Inappropriate prescribing and adverse drug events in older people. *BMC Geriatr*. Jan 28 2009;9:5. doi:10.1186/1471-2318-9-5
11. Wu C, Bell CM, Wodchis WP. Incidence and economic burden of adverse drug reactions among elderly patients in Ontario emergency departments: a retrospective study. *Drug Saf*. Sep 1 2012;35(9):769-81. doi:10.1007/bf03261973
12. Scott IA, Hilmer SN, Reeve E, et al. Reducing inappropriate polypharmacy: the process of deprescribing. *JAMA Intern Med*. May 2015;175(5):827-34. doi:10.1001/jamainternmed.2015.0324
13. Levinson W. Choosing Wisely Canada Recommendations. November 12, 2018. Accessed November 12, 2018. https://choosingwiselycanada.org/recommendations/
14. O'Mahony D, O'Sullivan D, Byrne S, O'Connor MN, Ryan C, Gallagher P. STOPP/START criteria for potentially inappropriate prescribing in older people: version 2. *Age Ageing*. Mar 2015;44(2):213-8. doi:10.1093/ageing/afu145
15. American Geriatrics Society 2015 Updated Beers Criteria for Potentially Inappropriate Medication Use in Older Adults. *J Am Geriatr Soc*. Nov 2015;63(11):2227-46. doi:10.1111/jgs.13702
16. Cross C. Introducing deprescribing into culture of medication. *Cmaj*. Sep 17 2013;185(13):E606. doi:10.1503/cmaj.109-4554
17. Frank C. Deprescribing: a new word to guide medication review. *Cmaj*. Apr 1 2014;186(6):407-8. doi:10.1503/cmaj.131568

18. Gallagher P, Lang PO, Cherubini A, et al. Prevalence of potentially inappropriate prescribing in an acutely ill population of older patients admitted to six European hospitals. *Eur J Clin Pharmacol*. Nov 2011;67(11):1175-88. doi:10.1007/s00228-011-1061-0
19. Forster AJ, Clark HD, Menard A, et al. Adverse events among medical patients after discharge from hospital. *Cmaj*. Feb 3 2004;170(3):345-9.
20. Counter D, Millar JWT, McLay JS. Hospital readmissions, mortality and potentially inappropriate prescribing: a retrospective study of older adults discharged from hospital. *Br J Clin Pharmacol*. Aug 2018;84(8):1757-1763. doi:10.1111/bcp.13607
21. Woolcott JC, Richardson KJ, Wiens MO, et al. Meta-analysis of the impact of 9 medication classes on falls in elderly persons. *Arch Intern Med*. Nov 23 2009;169(21):1952-60. doi:10.1001/archinternmed.2009.357
22. Steinman MA, Rosenthal GE, Landefeld CS, Bertenthal D, Kaboli PJ. Agreement between drugs-to-avoid criteria and expert assessments of problematic prescribing. *Arch Intern Med*. Jul 27 2009;169(14):1326-32. doi:10.1001/archinternmed.2009.206
23. Potter K, Flicker L, Page A, Etherton-Beer C. Deprescribing in Frail Older People: A Randomised Controlled Trial. *PLoS One*. 2016;11(3):e0149984. doi:10.1371/journal.pone.0149984
24. Gillespie U, Alassaad A, Henrohn D, et al. A comprehensive pharmacist intervention to reduce morbidity in patients 80 years or older: a randomized controlled trial. *Arch Intern Med*. May 11 2009;169(9):894-900. doi:10.1001/archinternmed.2009.71
25. Pellegrin KL, Krenk L, Oakes SJ, et al. Reductions in Medication-Related Hospitalizations in Older Adults with Medication Management by Hospital and Community Pharmacists: A Quasi-Experimental Study. *J Am Geriatr Soc*. Jan 2017;65(1):212-219. doi:10.1111/jgs.14518
26. Iyer S, Naganathan V, McLachlan AJ, Le Couteur DG. Medication withdrawal trials in people aged 65 years and older: a systematic review. *Drugs Aging*. 2008;25(12):1021-31. doi:10.2165/0002512-200825120-00004
27. Reeve E, Thompson W, Farrell B. Deprescribing: A narrative review of the evidence and practical recommendations for recognizing opportunities and taking action. *Eur J Intern Med*. Mar 2017;38:3-11. doi:10.1016/j.ejim.2016.12.021
28. Thillainadesan J, Gnjidic D, Green S, Hilmer SN. Impact of Deprescribing Interventions in Older Hospitalised Patients on Prescribing and Clinical Outcomes: A Systematic Review of Randomised Trials. *Drugs Aging*. Apr 2018;35(4):303-319. doi:10.1007/s40266-018-0536-4
29. Leape LL, Bates DW, Cullen DJ, et al. Systems analysis of adverse drug events. ADE Prevention Study Group. *Jama*. Jul 5 1995;274(1):35-43.
30. McDonald EG, Wu PE, Rashidi B, et al. The MedSafer Study: A Controlled Trial of an Electronic Decision Support Tool for Deprescribing in Acute Care. *J Am Geriatr Soc*. Sep 2019;67(9):1843-1850. doi:10.1111/jgs.16040
31. Tamblyn R, Abrahamowicz M, Buckeridge DL, et al. Effect of an Electronic Medication Reconciliation Intervention on Adverse Drug Events: A Cluster Randomized Trial. *JAMA Netw Open*. Sep 4 2019;2(9):e1910756. doi:10.1001/jamanetworkopen.2019.10756
32. Yu L, Buysse DJ, Germain A, et al. Development of short forms from the PROMIS sleep disturbance and Sleep-Related Impairment item banks. *Behav Sleep Med*. Dec 28 2011;10(1):6-24. doi:10.1080/15402002.2012.636266
33. Herdman M, Gudex C, Lloyd A, et al. Development and preliminary testing of the new five-level version of EQ-5D (EQ-5D-5L). *Qual Life Res*. Dec 2011;20(10):1727-36. doi:10.1007/s11136-011-9903-x
34. Cummings P. Estimating adjusted risk ratios for matched and unmatched data: An update. Accessed May 4, 2021. https://journals.sagepub.com/doi/pdf/10.1177/1536867X1101100208

35. Xie F, Pullenayegum E, Gaebel K, et al. A Time Trade-off-derived Value Set of the EQ-5D-5L for Canada. *Med Care*. Jan 2016;54(1):98-105. doi:10.1097/mlr.0000000000000447
36. Barker D, D'Este C, Campbell MJ, McElduff P. Minimum number of clusters and comparison of analysis methods for cross sectional stepped wedge cluster randomised trials with binary outcomes: A simulation study. *Trials*. 2017;18(1):119-119. doi:10.1186/s13063-017-1862-2
37. Thompson JA, Davey C, Fielding K, Hargreaves JR, Hayes RJ. Robust analysis of stepped wedge trials using cluster-level summaries within periods. *Stat Med*. Jul 20 2018;37(16):2487-2500. doi:10.1002/sim.7668
38. Thompson J, Davey C, Hayes R, Hargreaves J, Fielding K. swpermute: Permutation tests for Stepped-Wedge Cluster-Randomised Trials. *Stata J*. Dec 1 2019;19(4):803-819. doi:10.1177/1536867x19893624
39. Forster AJ, Asmis TR, Clark HD, et al. Ottawa Hospital Patient Safety Study: incidence and timing of adverse events in patients admitted to a Canadian teaching hospital. *Cmaj*. Apr 13 2004;170(8):1235-40.
40. Forster AJ, Murff HJ, Peterson JF, Gandhi TK, Bates DW. The incidence and severity of adverse events affecting patients after discharge from the hospital. *Ann Intern Med*. Feb 4 2003;138(3):161-7.
41. Hemming K. A menu-driven facility for power and detectable-difference calculations in stepped-wedge cluster-randomized trials. *Stata Journal*. 2014;2(14):363-380.
42. Hussey MA, Hughes JP. Design and analysis of stepped wedge cluster randomized trials. *Contemp Clin Trials*. Feb 2007;28(2):182-91. doi:10.1016/j.cct.2006.05.007

Section 18: Sample screenshots from the MedSafer Application

Patient list (fake names):

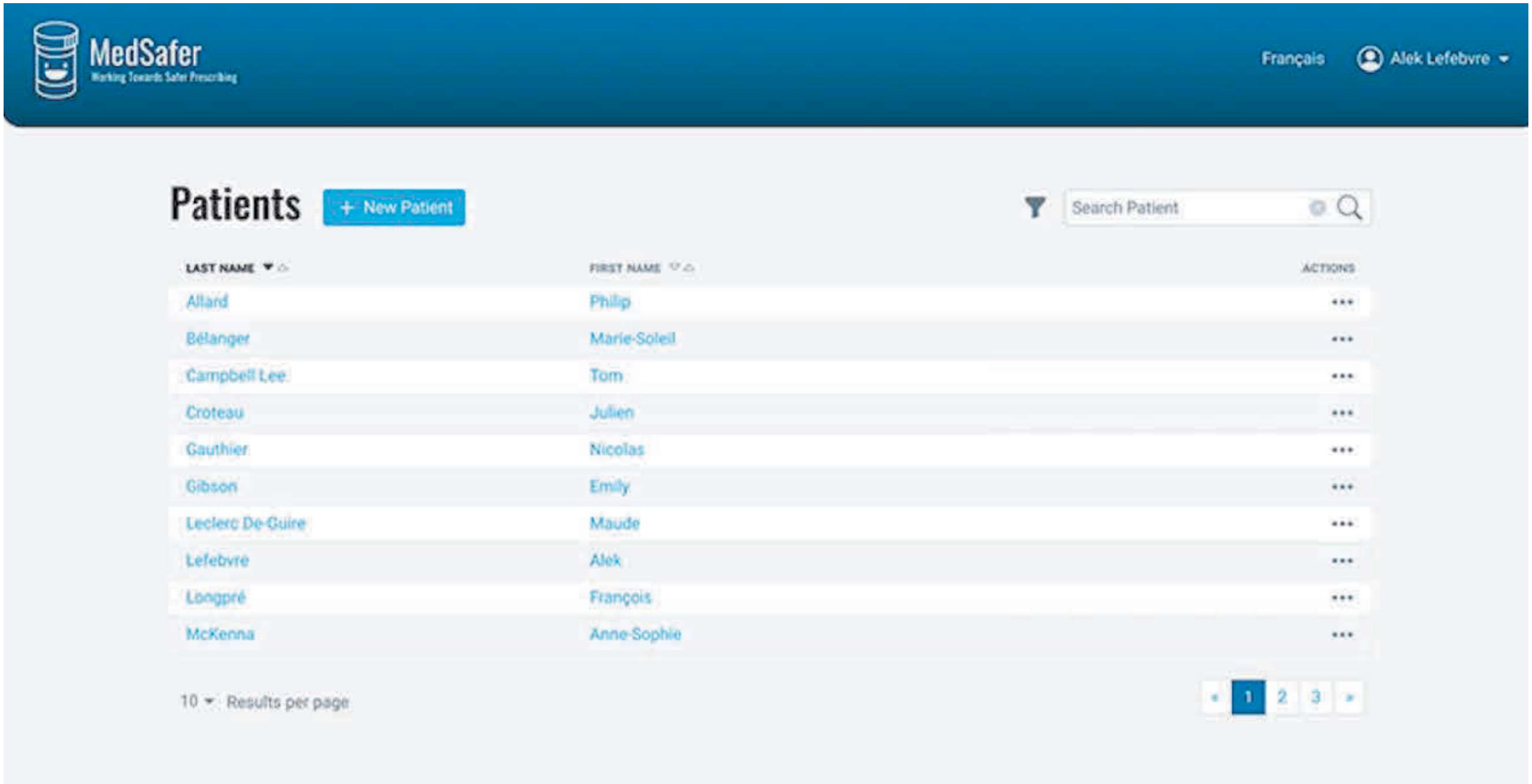

Patient Medical Conditions:

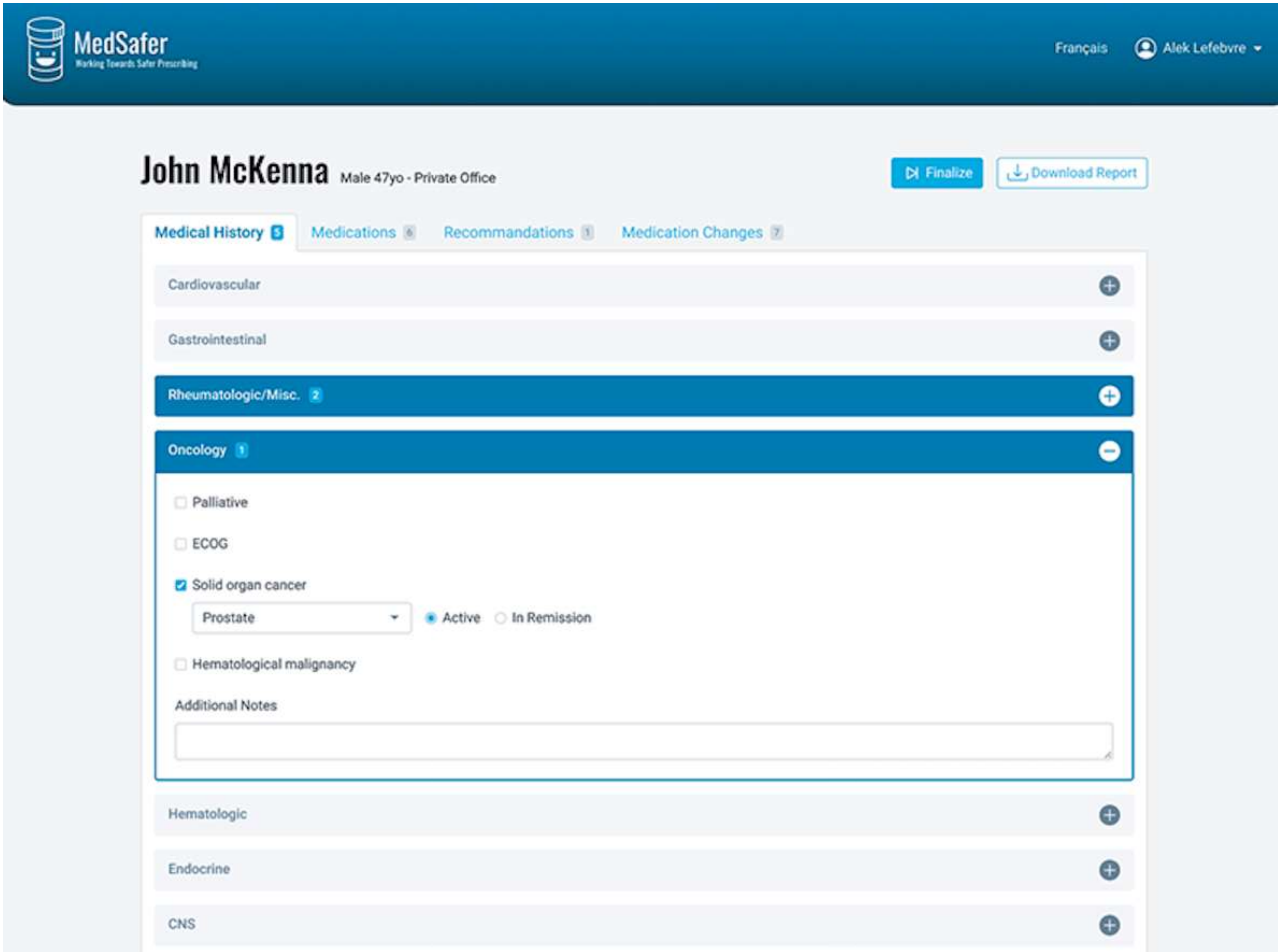

Medications:

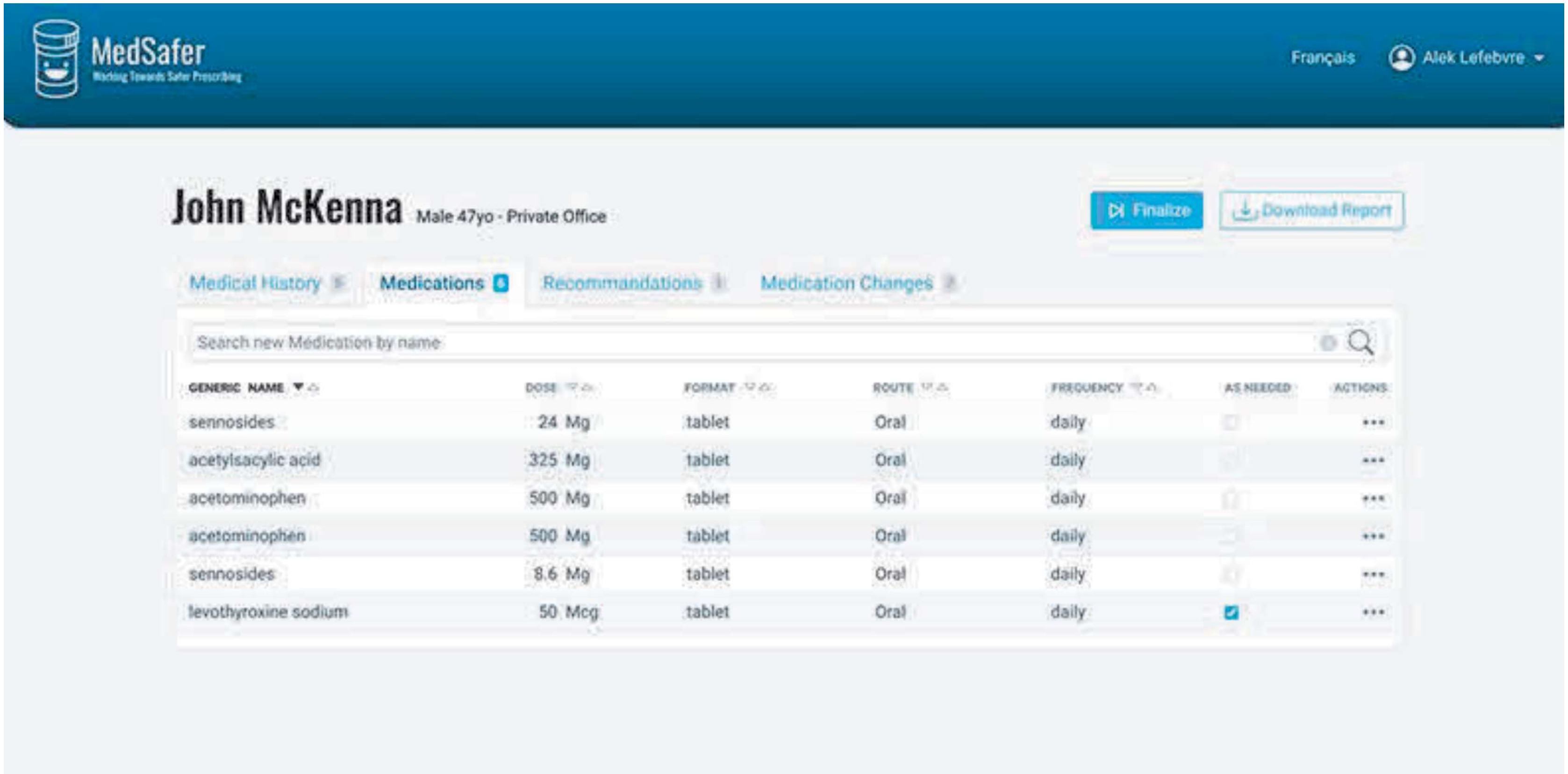

Recommendations:

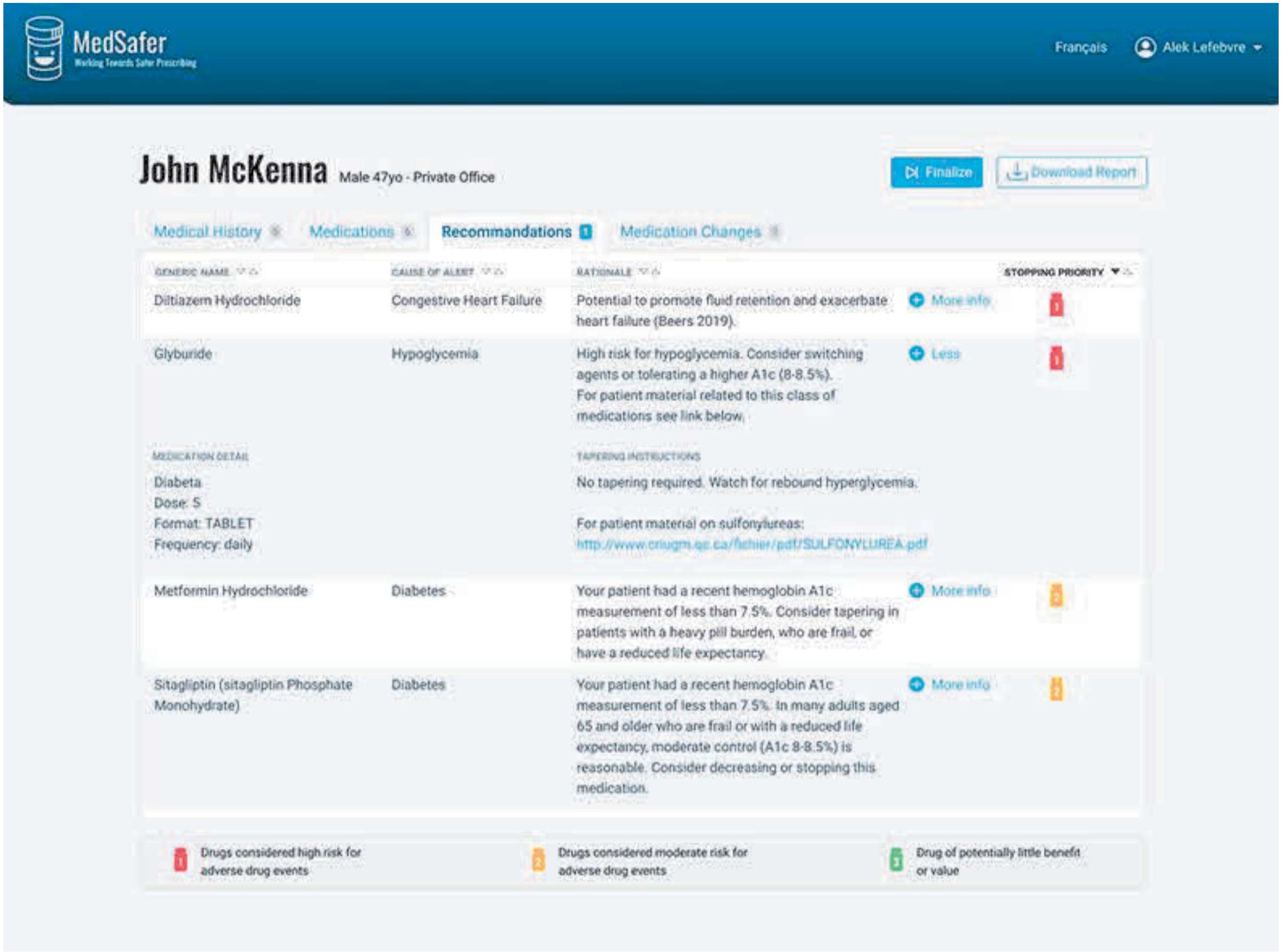

Supplement: Supplement 1. — Trial Protocol [file jamainternmed-e217429-s001.pdf]
