# Supplementary material for: The MedSafer Study—Electronic Decision Support for Deprescribing in Hospitalized Older Adults: A Cluster Randomized Clinical Trial
Source: JAMA Intern Med. 2022 Jan 18;182(3):1–10. doi: 10.1001/jamainternmed.2021.7429 (PMC8767487; doi:10.1001/jamainternmed.2021.7429)
Supplement: Supplement 2. — eMethods. Table of Contents and Section A-I eTable 1. Details of the Timing of Deprescribing Report Reviews eTable 2. Additional Details of Academic Detailing by Site eFigure 1. Study Design Diagram eFigure 2. ADWEs are a subcategory of all ADEs eTable 3. Classification of Adverse Drug Events by Intervention Status eTable 4. Sensitivity Analysis for the Primary Outcome of 30-day Adverse Drug Events that Includes Grade 4 Possible Adverse Drug Events (ADE) eFigure 3. Subgroup effects by Intervention Status for the Primary Outcome of 30-day Adverse Drug Events eTable 5. PROMIS Sleep Disturbance Score Pre- and Post-hospitalization by Intervention Status eTable 6. Sleep Quality Pre- and Post-hospitalization by Intervention Status eTable 7. Sleep is Refreshing Pre- and Post-hospitalization by Intervention Status eTable 8. Trouble Sleeping Pre- and Post-hospitalization by Intervention Status eTable 9. Difficulty Falling Asleep Pre- and Post-hospitalization by Intervention Status eTable 10. Quality of Life by Intervention Status eFigure 4. Subgroup Effects by Intervention Status for the Secondary Outcome of Proportion with 1 or more Potentially Inappropriate Medications Deprescribed eFigure 5. Unadjusted Number of PIMs Stopped in Control vs Intervention eTable 11. Sensitivity Analyses [file jamainternmed-e217429-s002.pdf]

Supplemental Online Content

McDonald EG, Wu PE, Rashidi B, et al. The MedSafer study—electronic decision support for deprescribing in hospitalized older adults: a cluster randomized clinical trial. *JAMA Intern Med*. Published online January 18, 2021. doi:10.1001/jamainternmed.2021.7429

eMethods. Table of Contents and Sections A-I
eTable 1. Details of the Timing of Deprescribing Report Reviews
eTable 2. Additional Details of Academic Detailing by Site
eFigure 1. Study Design Diagram
eFigure 2. ADWEs are a subcategory of all ADEs
eTable 3. Classification of Adverse Drug Events by Intervention Status
eTable 4. Sensitivity Analysis for the Primary Outcome of 30-day Adverse Drug Events that Includes Grade 4 Possible Adverse Drug Events (ADE)
eFigure 3. Subgroup effects by Intervention Status for the Primary Outcome of 30-day Adverse Drug Events
eTable 5. PROMIS Sleep Disturbance Score Pre- and Post-hospitalization by Intervention Status
eTable 6. Sleep Quality Pre- and Post-hospitalization by Intervention Status
eTable 7. Sleep is Refreshing Pre- and Post-hospitalization by Intervention Status
eTable 8. Trouble Sleeping Pre- and Post-hospitalization by Intervention Status
eTable 9. Difficulty Falling Asleep Pre- and Post-hospitalization by Intervention Status
eTable 10. Quality of Life by Intervention Status
eFigure 4. Subgroup Effects by Intervention Status for the Secondary Outcome of Proportion with 1 or more Potentially Inappropriate Medications Deprescribed
eFigure 5. Unadjusted Number of PIMs Stopped in Control vs Intervention
eTable 11. Sensitivity Analyses

This supplemental material has been provided by the authors to give readers additional information about their work.

© 2021 McDonald EG et al. *[JAMA Internal Medicine]*.

eMethods.

Section A	• Inclusion and exclusion criteria<br>• Requirements to use the software and how to access the software<br>• How to deprescribe without the software (a step-by-step guide)
Section B	Intervention implementation at each hospital site
Section C	Additional Cluster Details
Section D	Additional details of adjudication and adverse drug events
Section E	Sample pamphlet on deprescribing
Section F	Sample MedSafer report
Section G	Sample deprescribing report
Section H	Supplemental Analyses:<br>• Commonly alerted PIMs (eTable 1)<br>• Adverse Drug Event Classification (eTable 2)<br>• Sensitivity analysis for grade 4 ADEs and higher (eTable 3)<br>• Impact on Sleep and Quality of Life (eTables 4-9)<br>• Post Discharge Emergency Room Visits/Hospitalizations<br>• Post Discharge Deaths<br>• Post Discharge Falls<br>• Absolute number of medications at discharge<br>• Absolute number of PIMs at discharge<br>• Absolute number of medications at 30 days post-discharge<br>• Subgroup effects for ADEs looking at effect by sex, by frailty, by long term care status, by palliative status (eFigure 1)<br>• Subgroup effects for deprescribing looking at effect by sex, by frailty, by long term care status, by palliative status (eFigure 2)
Section I	Post-Hoc Analyses

© 2021 McDonald EG et al. *[JAMA Internal Medicine].*

Section A: Inclusion and Exclusion Criteria and Additional Details

Specific inclusion criteria:

Participants eligible for this study include patients who are i) aged 65 years or older; ii) taking 5 or more medications; and iii) admitted to the study units.

Subject exclusion criteria:

- Expected to die within 1-3 months
- Patient or proxy do not speak English or French
- Did not provide consent
- No provincial health insurance
- Will be unable to reach by telephone post-discharge
- Taking fewer than five usual home medications
- Admission or transfer to a non-study unit is expected: patients who are ultimately discharged from non-study units during their hospitalization will be excluded unless that unit is a transitional care, rehabilitation, or post-acute care unit used to bridge the gap between acute medical hospitalization and community services. For example, patients transferred to and subsequently discharged from a surgical unit (where there is no intervention) will need to be excluded.

Requirements to use the software:

1. MedSafer software exists in 4 different formats. To learn more about accessing the software, please visit medsafer.org.
    a. A research format (used for this study)
        i. A webpage that is accessed by the research assistant with a password secured login
        ii. Details about the participant are collected by the research assistant and manually entered into the online webpage; these include demographics, contact information, consent details, medical comorbidities and medications.
        iii. Comorbidities are manually checked off (check boxes)
        iv. Medications are typed in using a search function and drop down menu
        v. The report is automatically generated by clicking on "generate report" and is available in the webpage for visualization and as a downloadable PDF
        vi. Medication outcomes (stopped, modified or continued) are entered manually into the application
        vii. A module for collecting details about adverse drug events post discharge is available for an interviewer
        viii. A module for reviewers to adjudicate adverse drug events is available
        ix. Manual data entry is required; each entry takes about 7-8 minutes for a trained professional
    b. A "public facing" version (can be accessed by healthcare professionals)
        i. Same as the research format but requires fewer data entry points

© 2021 McDonald EG et al. *[JAMA Internal Medicine].*

ii. Requires manual data entry into the webpage (check boxes and drop-down menus)
iii. Available for immediate use by contacting the study team for a small annual fee

c. A "patient facing" version (can be accessed by patients or families)
   i. A simplified version with patient friendly prompts, non-expert language that produces a report that can be taken to the person's healthcare professional or provides a link for the healthcare professional to log in and view results
   ii. Some medications that are low risk are directly displayed to the used with deprescribing instructions (e.g., docusate, NSAIDs, sleeping pills) and links to the Canadian Deprescribing Network EMPOWER brochures.
   iii. Requires manual data input

ci. An application programming interface
   i. Analyzes coded data from the electronic medical record in the format of drug identification numbers, AHFS classification, etc… and ICD 10 codes
   ii. No manual data input required
   iii. Accessible by contacting the study team but requires an annual fee and set-up/development costs are associated
   iv. Currently available and used in Ontario and New Brunswick long term care homes

To deprescribe without the use of technology the following steps can be followed:

- An expert clinician in deprescribing (geriatrician, internist, generalist with training, pharmacist etc…)
- Access to existing deprescribing guidelines (static versions exist in print and online; we use the criteria from the American Geriatrics Society, Choosing Wisely Canada and STOPP criteria). References for these documents are available in the main document
- An accurate up-to-date list of medications (mediation reconciliation)
- An up-to-date list of medical conditions
- Cross reference medications, medical conditions, and guidelines to generate a list of medications for deprescribing
- Discuss with the person their values and preferences when going over the deprescribing plan
- Access medstopper.com to ensure that no drugs cause rebound or need tapering
- Implement the deprescribing plan
- Print out EMPOWER brochures from the Canadian Deprescribing Network (available for some medications) to increase patient and provider involvement
- Monitor person throughout deprescribing plan
- Readjust deprescribing plan as needed
- Steps in a deprescribing plan or process have been previously described by Scott et al., (as well as others); https://jamanetwork.com/journals/jamainternalmedicine/article-abstract/2204035.

Additional details of the intervention:

© 2021 McDonald EG et al. *[JAMA Internal Medicine].*

- **Medical teams** were **aware of an ongoing research study** into post-discharge adverse events but were blinded to the specific study goals to minimize any changes in practice due to observation.
- **Patients** were told that a study was taking place whereby the treating team would **review their medications** (standard care) and that as part of this process the team might make suggestions that could stop or reduce certain medications or suggest switching to a safer class of medication based on their age, medical conditions, prognosis and/or interactions with other medications they were taking. Patients consented to a phone call at 30-days to see how they were affected by the intervention following their hospital discharge.
- Units that had participated in the **previous pilot study** had at least a 3-month wash-out period during which no deprescribing reports were provided.
- **Admission notes** contain a detailed interview with the patient and/or proxy and a review of the medical record.
- The **Best Possible Medication History** (BPMH) was performed by a pharmacy team member and entailed a structured interview with the patient and/or proxy, listing all medications, whether they were taken as prescribed, and any side effects.
- **Changes to home medications** at discharge that were captured and categorized included new starts, change in dose or class, tapering or discontinuation.
- **Pharmacy support**: All but one study unit had access to dedicated pharmacy team members (pharmacists and/or pharmacy technicians) during usual business hours.
- **Medication counts**: we excluded topical preparations and ophthalmic solutions.

© 2021 McDonald EG et al. *[JAMA Internal Medicine]*.

Section B: Intervention Implementation at Each Hospital Site:

eTable 1: Details of the Timing of Deprescribing Report Reviews

**Hospital**	**Language**	**Medication reconciliation performed by:**	**Report display form**	**Timing of review**
Royal Victoria Hospital	EN/FR	Pharmacist	Printed report to team	At sign out*
Montreal General Hospital	EN/FR	Pharmacist	Printed report to team and in chart	At sign out
Lachine Hospital	FR/EN	Pharmacy technician	Printed in chart	By MD during daily progress notes
Toronto Western	EN	Pharmacist	Report emailed to staff and team. Printed for pharmacy	At sign out
Toronto General	EN	Pharmacist or pharmacy technician	Printed in chart; emailed/texted to medical blackberry	During rounds with pharmacy
The Ottawa Hospital: General campus	EN/FR	Pharmacist	Displayed in EMR	During chart review & pharmacy rounds
The Ottawa Hospital: The Civic campus	EN/FR	Pharmacist	Displayed in EMR	During chart review & pharmacy rounds
Kingston General Hospital	EN	Pharmacist	Printed in chart	Sign out
Foothills Medical Centre	EN	Pharmacist	Printed in chart	During rounds with pharmacy
Edmonton	EN	Pharmacist	Printed in chart	Reviewed by pharmacist
St. Paul’s	EN	Pharmacist	Printed in chart	Sign out

EN=English; FR=French; EMR=electronic medical record

** Sign out refers to when the clinical team (usually medical students, residents, and the attending physician) meets at the end of the day and go through the list of patients admitted to the medical unit one at a time and update each other and/or make a plan for the following day. New admissions and planned discharges are also discussed. Teaching may take place.*

© 2021 McDonald EG et al. *[JAMA Internal Medicine]*.

Additional academic detailing and quality improvement interventions to promote uptake by site:

eTable 2: Additional Details of Academic Detailing by Site

**Hospital**	**Quality improvement interventions/additional academic detailing**
Royal Victoria Hospital	Posters, buttons, stickers, medical grand rounds, direct teaching to pharmacists, email communique to physicians
Montreal General Hospital	Posters, buttons, stickers, medical grand rounds, teaching with ward-based nurses, direct teaching to pharmacists, email communique to physicians
Lachine Hospital	Posters, buttons, stickers, medical grand rounds, email communique to physicians
Toronto Western	Posters, buttons, stickers, medical grand rounds, monthly attending meeting, medical grand rounds
Toronto General	Posters, buttons, stickers, medical grand rounds, direct teaching with team pharmacists, monthly attending meeting, monthly quality improvement rounds, emails to staff physicians and team devices
The Ottawa Hospital: General campus	Posters, buttons, stickers, medical grand rounds, internal newsletter, resident orientation
The Ottawa Hospital: Civic campus	Posters, buttons, stickers, medical grand rounds, internal newsletter, resident orientation
Kingston General Hospital	Posters, buttons, stickers, presentation to residents, local resident champion
Foothills Medical Centre	Posters, buttons, stickers, communication to residents and staff,
Edmonton	Posters, buttons, stickers, direct pharmacy teaching
St. Paul’s	Posters, buttons, stickers, monthly attending rounds

© 2021 McDonald EG et al. *[JAMA Internal Medicine].*

Study Design:

eFigure 1: Study Design Diagram

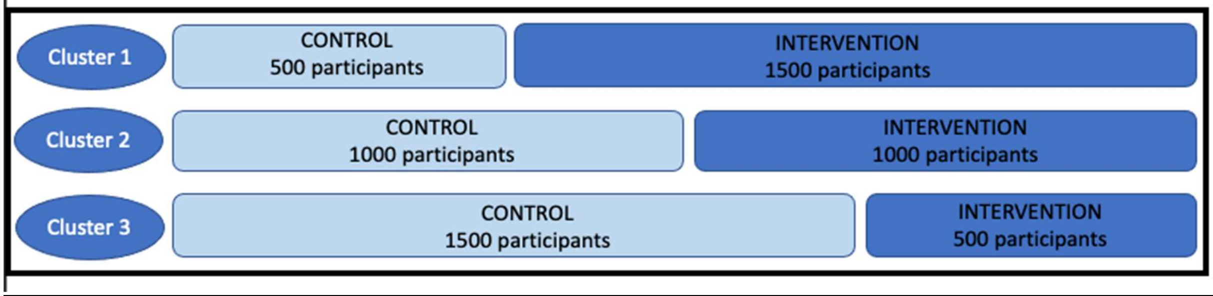

Study Units:

Study units were:

- Medical (surgical units were not included)
- Units were selected based on the presence of a pharmacist, the practice of medication reconciliation, if they were part of the study hospital where one of the study investigators practiced, internal medicine or general medicine.
- All units that were approached agreed to participate in the study
- Units were selected by the site co-investigator
- Units represent the largest clinical teaching units in Canada in four of the largest provinces (Quebec, Ontario, British Columbia and Alberta)
- Units are representative of typical academic medical unit across the country
- 1 community unit was included in order to increase the generalizability of the study

Interview details:

- The details of how the interviews were conducted are described in the MedSafer pilot as well as in the Study protocol
- Interviews were structured and the interviewer was blinded to the intervention status
- A structured case report form was filled out using an interview module that was developed for the MedSafer trial
- Patients who were readmitted to the hospital had their chart analyzed in detail and the reason for admission as well as the structured problem list were copied into the interview file and available to the adjudicator for determination of adverse events and adverse drug events.

Risk Classification of PIMs in Table 4 of the Main Manuscript

Examples of high-risk medications:
Combination blood thinners
Sedative hypnotics, benzodiazepines, trazodone, mirtazapine, or antipsychotics for sleep
Codeine and Tramadol
Opioids for chronic non-cancer pain

© 2021 McDonald EG et al. *[JAMA Internal Medicine].*

NSAIDs and a history of congestive heart failure

Examples of intermediate risk medications:
Proton pump inhibitors
Thiazides and a history of hyponatremia
Gabapentinoids
Diabetes therapy with a recent Hemoglobin A1C <7.5%
SSRIs and a history of recurrent falls

Examples of medications of little added value:
Docusate
Multiple daily doses of iron
Non-statin cholesterol lowering medications

Abbreviations:

PIM=potentially inappropriate medication
NSAIDs=nonsteroidal anti-inflammatory drugs
SSRIs=selective serotonin reuptake inhibitor

Breakdown of proportion of participants taking each PIM classification:

PIM category 1 (high risk): 2550/5345 (39.8%)
PIM category 2 (intermediate risk): 2661/5830 (43.5%)
PIM category 3 (little added value): 1277/2241 (16.7%)

© 2021 McDonald EG et al. *[JAMA Internal Medicine].*

Section C: Additional Cluster Details

Cluster 1 (Quebec): Royal Victoria Hospital (RVH), Montreal General Hospital (MGH), Lachine Hospital all in Montreal
Start date: RVH, MGH: August 22, 2017 Lachine: August 30, 2017
Intervention start date: February 26, 2018 all 3 sites
End date: September 13, 2019
Cluster 2 (Ontario): Toronto Western Hospital (TWH) & Toronto General Hospital (TGH), The Ottawa Hospital (TOH; Civic & General campuses), Kingston General Hospital (KGH)
Start date: TWH: Sep. 6, 2017, TGH: Sep. 8, 2017, TOH (both campuses): Sep. 19, 2017, KGH: Aug. 23-31, 2017 then resumed Feb. 7, 2018.
Intervention start date: TWH: Oct. 9, 2018, TGH: Nov. 27, 2018, TOH: Oct. 1, 2018, KGH: Dec. 3, 2018
End dates:
TWH: March 29, 2019 (break from Sept 5th, 2018 to Dec 2nd, 2018 due to no RA)
TGH: April 26, 2019 (break from Sept 15 - Nov 26, 2018 due to no RA)
TOH: Feb. 15, 2019 (both campuses)
KGH: May 5, 2019 (break from Sept 5th, 2018 to Dec 2nd, 2018 due to no RA)
Cluster 3 (West Coast): Foothills Medical Centre (Calgary), University of Alberta Hospital (Edmonton) and St. Paul's Hospital (Vancouver)
Start Date: Foothills: Sept. 19, 2017, University of Alberta: October 11, 2017, St. Paul's: October 25, 2017
Intervention start date: Foothills: Jan 21, 2019, University of Alberta: Jan 14, 2019 and St. Paul's: Jan 21, 2019
End date: Foothills: June 28, 2019 (loss of RAs), University of Alberta: Dec 13, 2019 and St. Paul's: Dec 13, 2019 (with break from May 4-June 13, 2018 due to no RA)

© 2021 McDonald EG et al. *[JAMA Internal Medicine]*.

Section D: Additional Details of Adjudication and Adverse Drug Withdrawal Events

Adjudication details:

Deliberate dose reductions: with respect to adverse drug event adjudication, dose reductions were not codified for adjudicators, but judgement was left up to the expert clinical discretion of the adjudicator (recall that all events were adjudicated in duplicate and resolved by a third independent reviewer in the case of disagreement).

- Adjudicators were provided with general guidelines (for example, small reductions in insulin were not to be considered clinically important or class changes between molecules etc...).
- In the adjudication form, adjudicators had access to the dose on arrival and at discharge and whether a taper had been ordered.
- Adjudicators did not have access to whether the dose reduction was deliberate per se (other than when a taper was ordered where this was evident) or whether the participant was in the intervention or control (blinded adjudication).

With respect to analysis, deprescribing or "deliberate dose reductions" were codified and analyzed blinded to the study intervention.

Proportion of medications that were stopped vs in the process of being deprescribed at the point of adjudication:

- Stopped medications: 76%
- "In tapering": 24%

eFigure 2: ADWEs are a subcategory of all ADEs

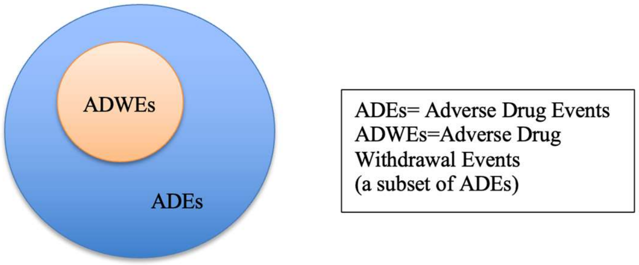

- ADWEs represented 49/249 ADEs (19.7%); the intervention led to a -0.1% (95%CI -1.2% to 1.0%) is the adjusted risk difference compared to the control.

© 2021 McDonald EG et al. *[JAMA Internal Medicine]*.

Section E: Sample Educational Pamphlet on Deprescribing

The pamphlet can be downloaded at https://pamphlet.medsafer.org

Section F: Sample EMPOWER brochures from the Canadian Deprescribing Network

Proton pump inhibitors: http://www.criugm.qc.ca/fichier/pdf/PPI-EN-Men.pdf

Sedative hypnotics/sleeping pills: http://www.criugm.qc.ca/fichier/pdf/BENZOeng.pdf

© 2021 McDonald EG et al. *[JAMA Internal Medicine]*.

Section G: Sample Deprescribing Opportunities Report

DEPRESCRIBING OPPORTUNITIES AS OF 2018-02-27

This document contains prioritized **opportunities** for a **reassessment** of the listed medications. Any decisions should take into context what you know about your patient and your clinical assessment of the **risks** and **benefits** of what has been presented.
If you have questions, suggestions, or you would like to report an error, please email support@medsafer.org with the subject line "MedSafer Study"

Name: Peter Pan 88Y
Neverland Hospital
MRN: *******
Born: 1929-xx-xx
Admission: 2016-xx-xx

TI - Tapering instructions or withdrawal concerns? / Please refer to tapering instructions

DRUGS CONSIDERED **HIGH RISK** FOR ADVERSE DRUG EVENT

Rule	Condition/Drug	Cause of Alert	Why might this be inappropriate?	TI
5	zopiclone (Imovane)		Don't use benzodiazepines or other sedative-hypnotics in older adults as first choice for insomnia, agitation, or delirium.	Yes
14	tiotropium-inh (Spiriva)	BPH	May worsen or induce urinary retention.	No
17	hydroxyzine (Atarax)		Highly anticholinergic; clearance reduced with advanced age, and tolerance develops when used as hypnotic; greater risk of confusion, dry mouth, constipation, and other anticholinergic effects and toxicity	Yes

DRUGS CONSIDERED **INTERMEDIATE RISK** FOR ADVERSE DRUG EVENT

Rule	Condition/Drug	Cause of Alert	Why might this be inappropriate?	TI
10	pantoprazole (Pantoloc)	Any	Chronic PPI therapy should be reevaluated regularly. For patients aged 60 years and older along with two or more of the following, ongoing therapy may be beneficial: antiplatelet, NSAID, systemic steroids, anticoagulation, prior upper gastrointestinal bleed. Other scenarios requiring ongoing therapy include: hypersecretory conditions, dual antiplatelet therapy, variceal banding within 14 days, and H. Pylori treatment.	Yes
50	mirtazapine (Remeron)	Any	Mirtazapine can be highly sedating and may increase the risk of falls and impaired cognition. This drug has reduced clearance in older adults. As first line treatment for sleep disorders try non-pharmacologic interventions to reduce the harms of overmedication.	Yes

© 2021 McDonald EG et al. *[JAMA Internal Medicine]*.

DRUGS OF **POTENTIALLY LITTLE BENEFIT OR VALUE**

Rule	Condition/Drug	Cause of Alert	Why might this be inappropriate?	TI
26	docusate-sodium (Colace)		Don't use stool softeners to prevent or treat constipation	No
60	calcium- carbonate (Oscal)	Any	There is little evidence that calcium supplementation is beneficial for fracture prevention (see JAMA. 2017;318 (24):2466-2482) and it may be associated with a higher risk of cardiovascular events. In general, calcium supplementation should be reserved for those with documented hypocalcemia.	No

© 2021 McDonald EG et al. *[JAMA Internal Medicine]*.

Tapering Instructions

IMOVANE ZOPICLONE
If the patient has been taking the medication for more than 3 months, a psychological dependence may have developed. In this case, consider tapering the drug over a period of 4 weeks, to improve the chances of stopping successfully.

ATARAX HYDROXYZINE
If used daily for more than 3-4 weeks, reduce dose by 50% every 1 to 2 weeks. Once at 25% of the original dose and no withdrawal symptoms have been seen, stop the drug. If any withdrawal symptoms occur, go back to approximately 75% of the previously tolerated dose.

PANTOLOC PANTOPRAZOLE
Stopping a PPI abruptly may lead to rebound hyperacidity. Patients on long-term therapy (>3 months) or high dose may benefit from tapering over 2-4 weeks. As needed H2 blocker therapy may mitigate some symptoms of rebound hyperacidity.

REMERON MIRTAZAPINE
Reduce dose by 25% every week. This can be extended or decreased (10% dose reductions) if needed. If intolerable withdrawal symptoms occur (usually 1-3 days after a dose change), go back to the previously tolerated dose and plan for a more gradual taper with the patient.

© 2021 McDonald EG et al. *[JAMA Internal Medicine]*.

Section H: Supplemental Prespecified Analyses

eTable 3: Classification of Adverse Drug Events by Intervention Status

Adverse Drug Event (N=249)	Total cohort N (%) N=249	Intervention N (%) N=111	Control N (%) N=138
Congestive heart failure/volume overload	43 (17.3)	18 (16.2)	25 (18.1)
Gastrointestinal bleed/hemorrhage/ hemoptysis/anemia/elevated INR	46 (18.5)	22 (19.8)	24 (17.4)
Hyperglycemia/hypoglycemia/uncontrolled blood glucose	8 (3.2)	3 (2.7)	5 (3.6)
Acute coronary syndrome/stroke/ atrial fibrillation/bradycardia/syncope/ rebound hypertension	18 (7.2)	6 (5.4)	12 (8.7)
Hypotension/orthostatic hypotension	18 (7.2)	11 (9.9)	7 (5.1)
Electrolyte abnormality/acute kidney injury	26 (10.4)	9 (8.1)	17 (12.3)
Weakness/falls	25 (10)	10 (9)	15 (10.9)
Opioid toxicity	4 (1.6)	2 (1.8)	2 (1.5)
Delirium	7 (2.8)	3 (2.7)	4 (2.9)
Pain/nausea/fatigue/other	50 (20.1)	26 (23.4)	24 (17.4)
Allergic reaction/hypersensitivity	4 (1.6)	1 (0.9)	3 (2.2)

© 2021 McDonald EG et al. *[JAMA Internal Medicine]*.

eTable 4: Sensitivity Analysis for the Primary Outcome of 30-day Adverse Drug Events that Includes Grade 4 Possible Adverse Drug Events (ADE)

**Cohort**	Period 1	Period 2	Period 3	Period 4	Total Control	Total Intervention
Cohort 1						
Number of Patients	426	467	465	508	1358	508
Adverse Drug Event	30 (7.0%)	32 (6.9%)	39 (8.4%)	33 (6.5%)	101 (7.4%)	33 (6.5%)
Any Adverse Event	125 (29.3%)	136 (29.1%)	153 (32.9%)	159 (31.3%)	414 (30.5%)	159 (31.3%)
Cohort 2						
Number of Patients	415	427	245	247	842	492
Adverse Drug Event	42 (10.1%)	38 (8.9%)	16 (6.5%)	25 (10.1%)	80 (9.5%)	41 (8.3%)
Any Adverse Event	147 (35.4%)	134 (31.4%)	65 (26.5%)	80 (32.4%)	281 (33.4%)	145 (29.5%)
Cohort 3						
Number of Patients	542	408	428	411	542	1247
Adverse Drug Event	39 (7.2%)	27 (6.6%)	29 (6.8%)	38 (9.3%)	39 (7.2%)	94 (7.5%)
Any Adverse Event	184 (34.0%)	130 (31.9%)	136 (31.8%)	114 (27.7%)	184 (34.0%)	380 (30.5%)
Intervention						
Number of Patients	--	408	673	1166	--	2247
Adverse Drug Event		27 (6.6%)	45 (6.7%)	96 (8.2%)		168 (7.5%)
Any Adverse Event	--	130 (31.9%)	201 (29.9%)	353 (30.3%)	--	684 (30.4%)
Pre-Intervention						
Number of Patients	1383	894	465	--	2742	--
Adverse Drug Event	111 (8.0%)	70 (7.8%)	39 (8.4%)		220 (8.0%)	
Any Adverse Event	456 (33.0%)	270 (30.2%)	153 (32.9%)	--	879 (32.1%)	--
**ADE Overall Adjusted Risk Difference = -2.3% (95% CI -4.9% to 0.4%)**<br>**Any Adverse Event Overall Adjusted Risk Difference = -1.2% (95% CI -6.4% to 4.1%)**<br>**Shaded areas represent intervention**						

© 2021 McDonald EG et al. *[JAMA Internal Medicine]*.

eTable 5-10: Pre- and Post-Hospitalization Sleep Scores by Intervention Status

eTable 5: PROMIS Sleep Disturbance Score Pre- and Post-hospitalization by Intervention Status

PROMIS Sleep Disturbance T-score		
	Pre-Hospital	Post Hospital
Control (n=2022)	52.4 (43.8-57.9)	48.4 (41.1-54.3)
Intervention (n=1601)	50.5 (43.8-57.9)	46.2 (41.1-54.3)

eTable 6: Sleep Quality Pre- and Post-hospitalization by Intervention Status

Sleep Quality				
	Prior to Hospital		Post Discharge	
	Control (n=2995)	Intervention (n=2249)	Control (n=2133)	Intervention (n=1762)
Very Good	384 (12.8)	261 (11.6)	325 (15.2)	243 (13.8)
Good	841 (28.1)	745 (33.1)	722 (33.9)	598 (33.9)
Fair	798 (26.6)	587 (26.1)	661 (31)	581 (33)
Poor	572 (19.1)	448 (19.9)	279 (13.1)	245 (13.9)
Very Poor	400 (13.4)	208 (9.3)	146 (6.8)	95 (5.4)

eTable 7: Sleep is Refreshing Pre- and Post-hospitalization by Intervention Status

Sleep is Refreshing				
	Prior to Hospital		Post Discharge	
	Control (n=2981)	Intervention (n=2226)	Control (n=2093)	Intervention (n=1695)
Very Much	303 (10.2)	195 (8.8)	355 (17.0)	276 (16.3)
Quite a bit	715 (24.0)	519 (23.3)	551 (26.3)	424 (25.0)
Somewhat	810 (27.2)	687 (30.9)	649 (31.0)	582 (34.3)
A little bit	478 (16.0)	346 (15.5)	297 (14.2)	221 (13.0)
Not at all	675 (22.6)	479 (21.5)	241 (11.5)	192 (11.3)

eTable 8: Trouble Sleeping Pre- and Post-hospitalization by Intervention Status

Have a Problem with Sleep				
	Prior to Hospital		Post Discharge	
	Control (n=2992)	Intervention (n=2232)	Control (n=2124)	Intervention (n=1735)
Not at all	881 (29.5)	858 (38.4)	877 (41.3)	816 (47.0)
A little bit	581 (19.4)	342 (15.3)	404 (19.0)	264 (15.2)
Somewhat	530 (17.7)	394 (17.7)	435 (20.5)	379 (21.8)
Quite a bit	522 (17.5)	411 (18.4)	252 (11.9)	166 (9.6)
Very much	478 (16.0)	227 (10.2)	156 (7.3)	110 (6.3)

© 2021 McDonald EG et al. *[JAMA Internal Medicine]*.

eTable 9: Difficulty Falling Asleep Pre- and Post-hospitalization by Intervention Status

Have difficulty falling asleep				
	Prior to Hospital		Post Discharge	
	Control (n=2991)	Intervention (n=2230)	Control (n=2124)	Intervention (n=1734)
Not at all	1416 (47.3)	1161 (52.1)	1351 (63.6)	1162 (67.0)
A little bit	463 (15.5)	312 (14.0)	233 (11.0)	200 (11.5)
Somewhat	449 (15.0)	309 (13.9)	259 (12.2)	185 (10.7)
Quite a bit	290 (9.7)	263 (11.8)	164 (7.7)	103 (5.9)
Very much	373 (12.5)	185 (8.3)	117 (5.5)	84 (4.8)

eTable 10: Quality of Life by Intervention Status

	EQ-5D-5L Derived QOL	Visual Analog Scale
Control (n=2122)	0.743 (0.425-0.871)	60 (50-75)
Intervention (n=1735)	0.722 (0.406-0.871)	60 (50-75)

Post Discharge Emergency Room Visits/Hospitalizations:

Control 459/2742 (16.7%) vs. Intervention 296/2247 (13.2%)
Adjusted OR 0.89 (95%CI 0.65-1.21)

Post Discharge Deaths:

Control 95/2742 (3.5%) vs. Intervention 75/2247 (3.3%)
Adjusted OR 1.13 (95%CI 0.65-2.01)

Post Discharge Falls:

Control 264/2742 (9.6%) vs. Intervention 207/2247 (9.2%)
Adjusted OR 0.76 (95%CI 0.57-1.05)

Absolute number of medications at discharge:

Intervention associated with -0.39 drugs (95%CI -0.65 to -0.14)

Absolute number of PIMs at discharge:

Intervention associated with -0.41 PIMS (95%CI -0.51 to -0.31)

Absolute number of medications at 30 days post-discharge:

Intervention associated with -0.09 drugs (95%CI -0.25 to 0.22)

© 2021 McDonald EG et al. *[JAMA Internal Medicine]*.

eFigure 3: Subgroup effects by Intervention Status for the Primary Outcome of 30-day Adverse Drug Events

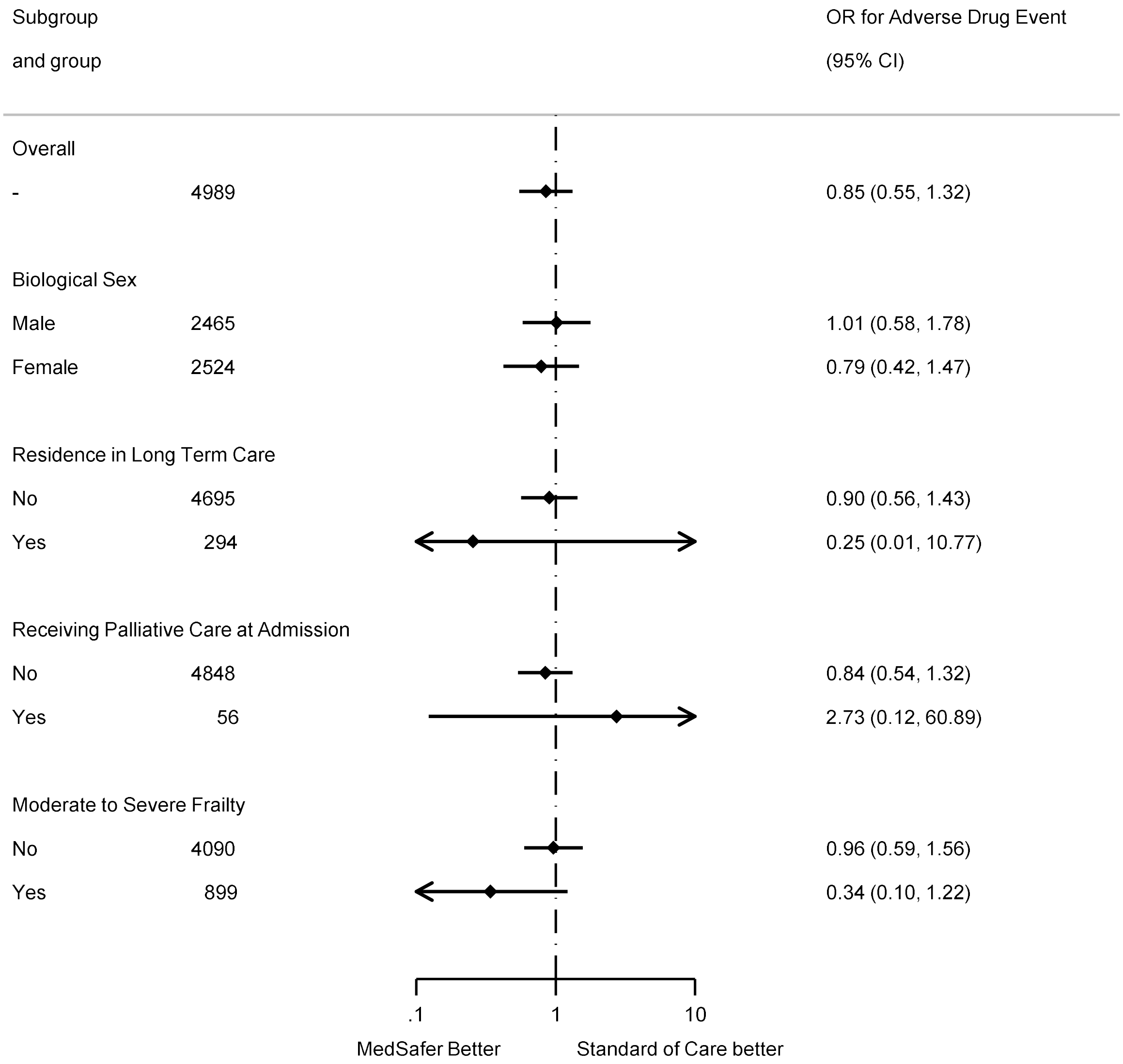

© 2021 McDonald EG et al. *[JAMA Internal Medicine]*.

eFigure 4: Subgroup Effects by Intervention Status for the Secondary Outcome of Proportion with 1 or more Potentially Inappropriate Medications Deprescribed

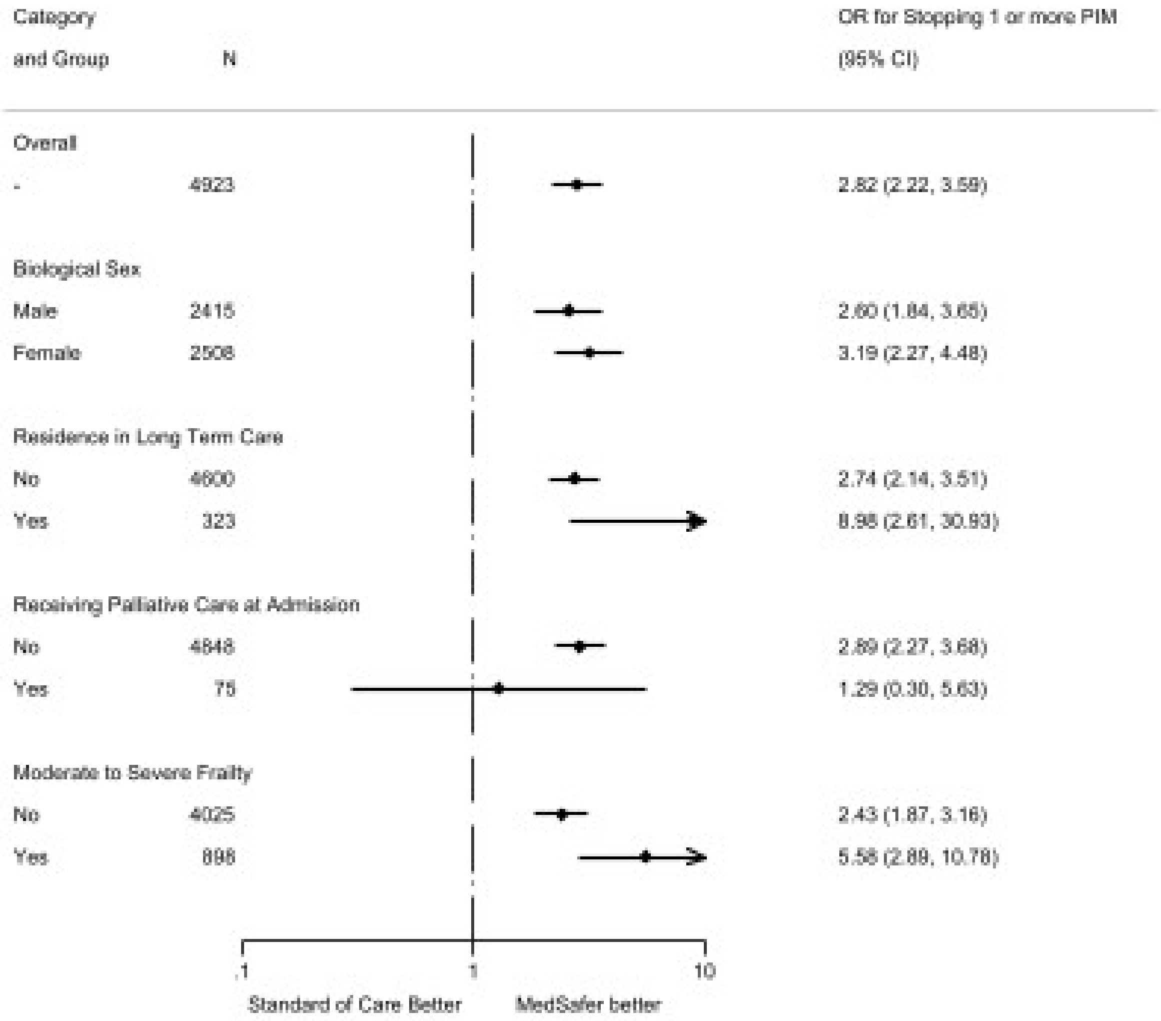

© 2021 McDonald EG et al. *[JAMA Internal Medicine]*.

Section I: Post-Hoc Sensitivity and Other Analyses Performed at the Request of the Editors and the Reviewers

1. Proportion of PIMs deprescribed
- Overall, control patients had 17% of their individual PIMs deprescribed compared to intervention patients who had 33.2% of their individual PIMs deprescribed.
- The overall proportion of individual PIMs deprescribed was 12.8% higher (95%CI 9.4%-16.3%) after adjustment for period, cluster and baseline number of PIMs in fixed effects.

eFigure 5: Unadjusted Number of PIMs Stopped in Control vs Intervention

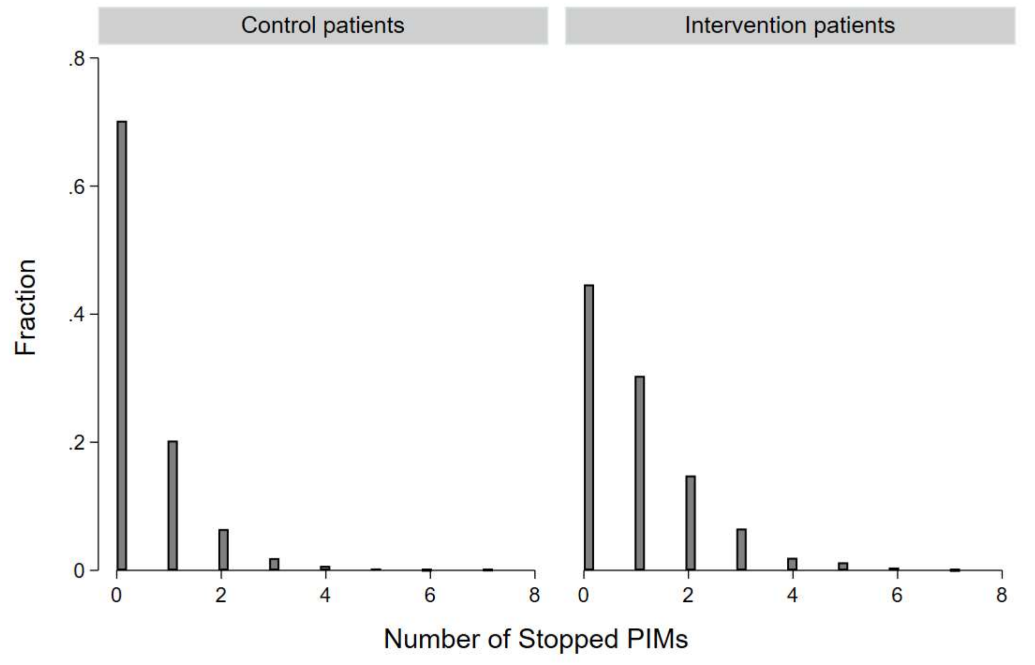

2. Post-hoc analysis adjusting for all values which differed by p<0.01
- Factors adjusted for: Age, English Language, Length of stay, Number of PIMs, Chronic Kidney Disease, Major Neurocognitive Disorder, Recurrent Falls and Moderate to Severe Frailty
- The adjusted analysis does not appreciably change the direction of effect or overall conclusions:
    - Adverse Drug Events: ARR -0.3% (95%CI -2.6% to 2.0%)
    - Adverse Events: ARR -0.5% (95%CI -4.7% to 3.5%)
    - Proportion with 1 or More PIMs Stopped: ARR 22.8% (95%CI 17.6% to 28.0%)

3. Sensitivity analyses that address low number of clusters in the study

eTable 11: Sensitivity Analyses

	Adverse Drug Event	Adverse Drug Event	Adverse Event	Adverse Event	Stopping 1 or more PIM	Stopping 1 or more PIM
Analysis	Adjusted Odds Ratio (95%CI)	Adjusted Risk Difference (95% CI)	Adjusted Odds Ratio (95%CI)	Adjusted Risk Difference (95% CI)	Adjusted Odds Ratio (95%CI)	Adjusted Risk Difference (95% CI)
Pre-specified	0.85 (0.55-1.32)	-0.8% (-2.9% to 1.3%)	0.95 (0.79-1.14)	-1.2% (-5.6% to 3.3%)	2.82 (2.22-3.59)	22.2% (16.9% to 27.4%)
Fixed effect by cluster	0.80 (0.49-1.32)	-1.0% (-3.3% to 1.3%)	0.92 (0.73-1.15)	-1.8% (-6.7% to 3.1%)	2.76 (2.18-3.51)	21.5% (16.5% to 26.5%)
Random effect by site	0.88 (0.59-1.32)	-0.6% (-2.5% to 1.3%)	0.94 (0.78-1.14)	-1.3% (-5.3% to 2.8%)	2.76 (2.17-3.52)	20.0% (14.9% to 25.1%)
Fixed effect by site	0.82 (0.50-1.34)	-0.9% (-3.3% to 1.4%)	0.91 (0.73-1.15)	-1.9% (-6.9% to 3.0%)	2.68 (2.10-3.42)	20.0% (15.1% to 24.9%)
Within period analysis by permutation*	--	-0.5% (-2.5% to 1.5%)	--	-1.1% (-3.3% to 1.1%)	--	37.9% (28.6% to 47.3%)

*Excludes periods 1 and 4 when all clusters/sites were in control and intervention, respectively

eTable 11 shows the results of four sensitivity analyses and allows the reader to compare to the pre-specified analysis. The sensitivity analyses are 1) a fixed effect by cluster (n=3); 2) a random effect by site (n=11); 3) a fixed effect by site (n=11); and 4) a non-parametric test (within period analysis by permutation).
