# Supplementary material for: The MedSafer Study—Electronic Decision Support for Deprescribing in Hospitalized Older Adults: A Cluster Randomized Clinical Trial
Source: JAMA Intern Med. 2022 Jan 18;182(3):1–10. doi: 10.1001/jamainternmed.2021.7429 (PMC8767487; doi:10.1001/jamainternmed.2021.7429)
Supplement: Supplement 3. — Data Sharing Statement [file jamainternmed-e217429-s003.pdf]

Data Sharing Statement

McDonald. The MedSafer Study-Electronic Decision Support for Deprescribing in Hospitalized Older Adults. *JAMA Intern Med*. Published January 18, 2022.
doi:10.1001/jamainternmed.2021.7429

Data
Data available: Yes
Data types: Deidentified participant data
How to access data: Request data from todd.lee@mcgill.ca
When available: beginning date: 09-01-2022, end date: 09-01-2023

Supporting Documents
Document types: None

Additional Information
Who can access the data: Researchers (non-industry funded) who request the data with an accompanying proposal for secondary analysis
Types of analyses: For a specified purpose
Mechanisms of data availability: With investigator support after approval of the proposal and with a signed data sharing agreement.
